# Morphology and life history divergence in cave and surface populations of *Gammarus lacustris* (L.)

**DOI:** 10.1371/journal.pone.0205556

**Published:** 2018-10-25

**Authors:** Kjartan Østbye, Eivind Østbye, Anne May Lien, Laura R. Lee, Stein-Erik Lauritzen, David B. Carlini

**Affiliations:** 1 Inland Norway University of Applied Sciences, Department of Forestry and Wildlife Management, Campus Evenstad, Koppang, Norway; 2 Center for Ecological and Evolutionary Synthesis (CEES), Department of Biosciences, University of Oslo, Oslo, Norway; 3 Knut Bjørhuus vei 35 C, Drammen, Norway; 4 Department of Biology, Stanford University, Stanford, CA, United States of America; 5 Department of Earth Science, University of Bergen, Allegaten 41, Bergen, Norway; 6 Department of Biology, American University, Washington, D.C., United States of America; Centre National de la Recherche Scientifique, FRANCE

## Abstract

Cave animals provide a unique opportunity to study contrasts in phenotype and life history in strikingly different environments when compared to surface populations, potentially related to natural selection. As such, we compared a permanent cave-living *Gammarus lacustris* (L.) population with two lake-resident surface populations analyzing morphology (eye- and antennal characters) and life-history (size at maturity, fecundity and egg-size). A part of the cytochrome *c* oxidase subunit I gene in the mitochondrion (COI) was analyzed to contrast genetic relationship of populations and was compared to sequences in GenBank to assess phylogeography and colonization scenarios. In the cave, a longer life cycle was implied, while surface populations seemed to have a shorter life cycle. Egg size, and size at maturity for both sexes, were larger in the cave than in surface populations, while fecundity was lower in the cave than in surface populations. The cave population had longer first- and second antennae with more articles, longer first- and second peduncles, and fewer ommatidia than surface populations. The cold low-productive cave environment may facilitate different phenotypic and life-history traits than in the warmer and more productive surface lake environments. The trait divergences among cave and surface populations resembles other cave-surface organism comparisons and may support a hypothesis of selection on sensory traits. The cave and Lake Ulvenvann populations grouped together with a sequence from Slovenia (comprising one genetic cluster), while Lake Lille Lauarvann grouped with a sequence from Ukraine (comprising another cluster), which are already recognized phylogenetic clusters. One evolutionary scenario is that the cave and surface populations were colonized postglacially around 9 000–10 000 years ago. We evaluate that an alternative scenario is that the cave was colonized during an interstadial during the last glaciation or earlier during the warm period before onset of the last glaciation.

## Introduction

Caves represent evolutionary model systems to study trait changes in the cave living fauna as compared to their relatives on the surface [1–3]. Striking apparent adaptations in morphology and life-history in cave animals have intrigued scientists for centuries [4–6]. Such cave dwelling populations, or species, often display trait reductions (often termed regressive evolution), reduced sight or even the complete loss of eyes and pigmentation, but attenuation of other appendages, as well as enhancement of extra-optic sensory structures, when compared to relatives outside the cave environment [7,8]. Since most obligate cave species (i.e. permanent residents of caves) often lack extant surface populations, or close taxonomic relatives, being separated for extended evolutionary time, they may not constitute the most ideal study objects for assessment of the very early stages of cave associated trait shifts and evolutionary changes. Facultative cave species (i.e. that also occupy surface areas) may better represent early stages in evolution of cave associated trait alterations where gene flow with surface populations still occurs or have recently been terminated [9–11]. The frequently observed infertility between cave and surface populations may also provide a possibility to analyze the genetic basis of complex traits [12,13]. Thus, cave animals are excellent model systems for studying speciation mechanisms at different stages in a long process [3,14–16].

Several hypotheses have been issued to test apparent evolution of trait divergence between cave and surface populations as seen in many organisms [7,17–19]. First, natural selection may reduce the economy allocated to an unused structure such as eyes in a dark and nutrient poor environment in caves to reallocate energy to more important traits. Alternatively, trait changes in surface and cave populations may be due to random genetic drift affecting evolutionary selected, neutral- or nearly neutral traits in small founder populations. Thus, trait changes may stem from different scenarios; (i) genetic drift on traits that are selected, neutral or nearly neutral in adaptation, (ii) single adaptive traits exposed to natural selection, (iii) correlated natural selection through simultaneous selection on two or more genetically independent traits, (iv) pleiotropy with selection on one single trait while other traits are tagging along due to genetic hitch-hiking and genomic linkage, or (v) a combination of these mechanisms, or other alternative phenotype-genotype mechanisms through gene expression pathways [6,12,20,21]. Here, phenotypic plasticity and epigenetics may also be important mechanisms in trait divergence in surface and cave environments, but have not yet been explored in much detail. At any rate, the apparent general similar selective pressures in caves seem to a large extent to result in remarkably similar evolutionary solutions to shared environmental challenges even for distantly related taxa [3,8,22].

In North America, the amphipod *Gammarus minus* has repeatedly colonized independent limestone caves from ancestral surface lake systems [19]. Here, traits usually do not differ between cave-dwelling and surface populations except for cave populations located in the Greenbrier Valley karst of West Virginia and in Tazewell County, Virginia. In these regions, cave populations are often highly modified phenotypically, have reduced eyes, fewer ommatidia, larger body size, longer antennae, and reduced pigmentation. Such morphological differences are attributed to natural selection [19,20,23,24], where genetic studies show that populations cluster based on hydrology rather than based on cave or surface environments [25]. Thus, for *G*. *minus*, troglomorphic characters have evolved independently in parallel due to repeated colonization of individuals of the surface populations dwelling at the spring where the cave water resurges [9]. Strong support for natural selection as a driver in phenotypic differences is observed in collapsed caves with *G*. *minus*, which then opens up a surface environment to the cave adapted populations, where subsequently natural selection towards functional eye structures evolves [19]. In a laboratory study, Fong [20] observed negative pleiotropy for reduction or loss of eye structures and increased antennae structures in cave-living *G*. *minus*, suggesting that one or both traits may be targeted by selection, while no negative pleiotropy was found within or between the surface populations of *G*. *minus*. Moreover, phylogenetic and population genetic studies often suggest that populations of cave species have a small effective population size (*N*_*e*_), lower genetic diversity, and reduced gene flow with their related surface populations [26–29]. Also, low genetic diversity and amino-acid codon usage bias may indicate bottlenecks, and further suggest constraint on adaptive evolution in cave population of *G*. *minus* [25].

A relative to the North American *Gammarus minus* is *G*. *lacustris* (G. O. Sars), which has a circumpolar distribution [30,31] from low altitude calcium-rich lakes to high altitude cold and often calcium-poor lakes [32]. The life-cycle in lowland areas are generally annual and semelparous (life histories characterized by death after first reproduction), while in colder habitats in the high mountain it comprises a multi-annual and iteroparous strategy (living to reproduce repeatedly), where two or three age classes are interpreted to occur simultaneously in the population [31,33–35]. *G*. *lacustris* is omnivorous, feeding on detritus, algae, fungi, and even conspecifics [36–41]. This species is also vulnerable to predation particularly from fish, but also from invertebrates [42–44]. Sexual dimorphism in body-size is observed, with males being larger than females [45]. The male likely has additional energetic costs when guarding the female by holding her in a firm grip (precopula) before the female molts and is fertilized [46,47], further suggesting that male body-size may be a trait targeted by both natural and sexual selection. After fertilization, the female keeps the eggs in her ventral brood pouch until release of the young [45). Natural selection for optimization of life histories also likely operates on the fecundity and egg-size correlations in female body-size in *Gammarus* sp. [31,43,48–50].

The recently deglaciated karst caves in Norway comprise a low-diversity cave fauna [51,52], almost lacking obligate cave dwellers, composing mostly facultative cave visitors. The low diversity of the cave-fauna in these northern areas is likely related to Weichselian ice-age cycles (the last glacial period and its associated glaciation is known in Northern Europe as the Weichselian glaciation) trough e.g. impaired colonization probabilities, and the rather young age of most of these caves [52]. The only “known” permanently resident limestone-cave population of *G*. *lacustris* is found in Southern Norway in the Sandågrotta Cave system [51]. This cave existed during the last ice-free period approximately 120 000 years before present (ybp) [51]. The late Weichselian ice sheet retreated from this area around 9–10 000 ybp [53], but signs of freshwater deposits stemming from an inter-glacial period is reported from the area [54,55]. These spatio-temporal environmental conditions suggest an extended putative temporal colonization window for *G*. *lacustris* into this area.

The aim of this study was to compare morphology and life history of the permanently cave-living population of *G*. *lacustris* with two lake-resident populations (hereafter termed cave and surface populations). In general, the cave environment can be described as cold, nutrient-poor, dark, and lacking predators (other than conspecifics), while the surface populations which lives in warmer, nutrient-rich habitats, have temporal changes in light-regimes due to winter-dependent ice-cover, and harbors a set of invertebrate (e.g. Odonata) and vertebrate predators (fish and birds). Our task was performed measuring a set of antennal and eye characters, along with life-history traits such as size-age classes based on temporal length distribution, size of maturity and female gonad investment in fecundity and egg size. To assess the genetic relationship among the three populations, we sequenced a part of the cytochrome *c* oxidase I gene (COI) encoded in mitochondrial DNA (mtDNA). A set of predictions was generated based on the general null-hypothesis that the cave population did not differ from the two surface populations either in traits or life history patterns. The evaluation for a rejection of this null-hypothesis would come from support of the following four predictions based on field observations: (i) the life-history cycle in the two environments is either shorter or longer due to more/less food, warmer/colder water and presence/absence of inter-specific predators, (ii) gonad allocation is higher or lower with regard to fecundity and egg size in the two contrasting environments–reflecting differential reproductive investment, (iii) antennae (first and second antennae) are smaller or larger in the two environments for search for mates and food items in light and dark environments, and (iv) eye traits (number of ommatidia and eye-area) in the two environments are reduced or increased as having lower or higher adaptive values in visible or dark environments. Furthermore, we discuss putative mechanisms behind our findings and also compare our results with similar pairs of surface and cave dwelling populations in the geographically and phylogenetically independent adaptive diversification seen in North American *G*. *minus* to evaluate if trait divergence and adaptation to cave and surface environments are replicated.

## Materials and methods

### Ethics regarding sampling

The crustacean Gammarus lacustris belongs to the taxonomic class Malacostraca (order Amphipoda) and is as such not underlying legislation with regard to ethics and sampling approval for scientific purposes in Norway. The sampling was conducted under an oral agreement and allowance from local landowners and County administration in municipality of Kongsberg. No written permit was needed. The cave environment is now protected by law.

### Study area

**Fig 1:**
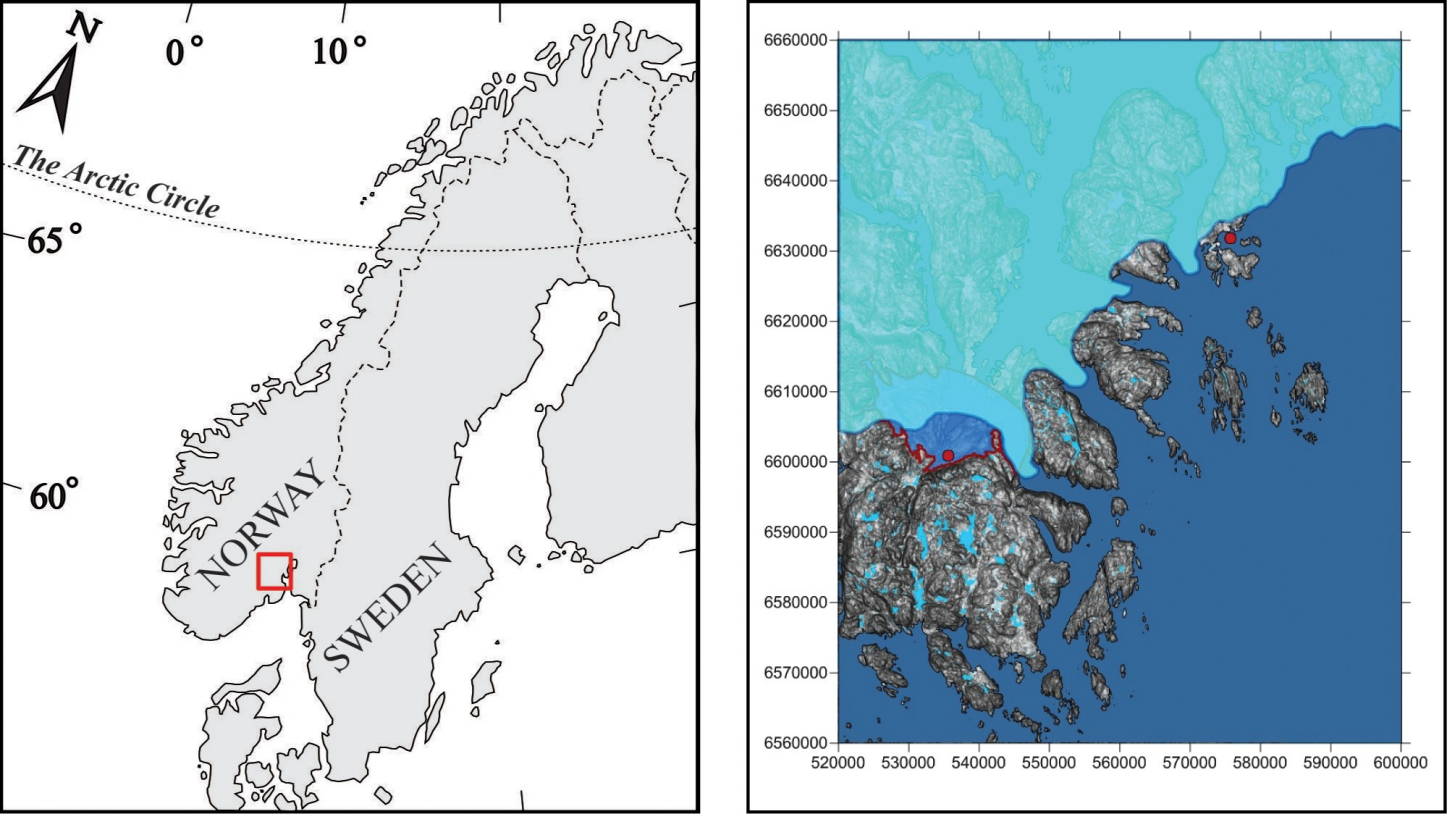
Study area and the estimated pro-glacial lake location. Left panel; map of Norway and study area. Right panel; the modelled position of the pro-glacial lake covering the Sandågrotta cave and Lake Lille Lauarvann (both situated in the red dot in lower part of figure). The geographically distant Lake Ulvenvann population is the other red dot in the upper part of figure. At this time, Lake Ulvenvann was not yet formed.

The Sandågrotta cave is situated on the Lauar plateau (59.32´ 34.90´´ latitude, 9.38´ 48.38´´ longitude), being a part of the northern foothill of the Skrimsfjella mountain ridge in the municipality of Kongsberg, in the county of Buskerud, southern Norway (Fig 1). To our knowledge, this cave harbours the only “known” permanent cave-living population of *G*. *lacustris*. The Lauar area completes the southwestern border of the geological province called the "Oslo-field” [56]. This region contains a complete Cambro-Silurian sequence of sediments, comprising lower Silurian reef and bioclastic limestones, in which the Sandågrotta cave system has developed. The cave area is situated at 370–400 m a.s.l. in a coniferous forest. There are several small lakes in the area, of which the nearest one, the lower situated Lake Lille Lauarvann at 343 m a.s.l. in an adjoining drainage system, was selected to compare the cave population with a closely situated surface population. In addition, we selected a population 100 km away, in the Lake Ulvenvannet, as a “replicate” of the surface environment type. This lake is situated at 180 m a.s.l. in the municipalities of Lier and Asker, in the counties of Akershus and Buskerud.

The Sandågrotta cave system has a length of more than 400 m. At present three larger parts of the system have been surveyed, reached by three separate entrances. Most of the system is water filled and has not yet been surveyed due to the inexplorable syphons (pers. comm. Norsk Teknisk Dykkerkrets). Here, *G*. *lacustris* inhabits the lower 2/3 of the system, composing a fairly large density in the calmer waterways, apparently avoiding the rapids in more river-like parts. The cave ponds are small, one to five m broad, up to 40 m long and with shallow maximum depths of maximum four meters. The bottom is composed of sediments of sand and gravel with scattered occurrence of stones in various size classes. Since this is an open cave system, the surface water entering the cave has a high influence on the underground water temperature, as well as entering organic matter, and makes it fluctuate during the year. Continuous monitoring of water temperature during the study period shows variation in mean temperature of 10.8 ^o^C in July to 2.0 ^o^C from December to April using a data logger (Grant D9B). The yearly mean water temperature was estimated to 4.4 ^o^C, and the accumulated yearly temperature-sum (degree-days; i.e. the yearly sum of degrees per day) was 1621 ^o^C degrees. This measure is a crude proxy for the environmentally dependent physiological scope for growth in ectothermic animals. In the cave environment, *G*. *lacustris* do not seem to have coexisting predator species, when considering either fish or invertebrate species except for potential cannibalism. This claim was supported by our lack of finding of such organisms in the traps and in the previous surveys of the cave environment over years.

The Lake Lille Lauarvann has an area of 0.2 km^2^ and is situated 600 m downhill from the Sandågrotta cave system (59.33´ 0.38´´latitude, 9.38´ 42.97´´longitude). It is an oligotrophic high-calcium content lake type called *Lobelia* lakes according to its algae vegetation that thrives in high calcium conditions. The highest monthly mean temperature, at 1.5 m depth, was in August with a temperature of 19.6 ^o^C. During the winter, from November to March, the temperature was not measured, but was assumed to be ca 4 ^o^C at a depth of 2 m, which is approximately the upper zone where *G*. *lacustris* has residence during winter. Thus, the yearly mean temperature was estimated to 9.3 ^o^C, with a temperature sum of 3407 ^o^C degrees. The fish fauna comprises brown trout (*Salmo trutta*), eurasian perch (*Perca fluviatilis*), minnow (*Phoxinus phoxinus*) and nine-spined stickleback (*Pungitius pungitius*). Thus, fish species predates on *G*. *lacustris*, as do invertebrate predators such as dragonfly larvae reflecting a high-predation environment as contrasted to the cave habitat population.

The second surface environment, Lake Ulvenvann is situated closer to the city of Oslo (59.48´ 56.6´´latitude, 10.21´ 1.14´´longitude) and has an area of 0.4 km^2^. This lake is mesotrophic with some interchanging bedrocks of calcium and granite, having a much lower calcium content than seen in both the Sandågrotta cave and Lake Lille Lauarvann. The temperature was not measured in this lake, but it was assumed to be higher than in Lake Lille Lauarvann due to its lower elevation. The fish fauna comprise perch, trout, minnow, crucian carp (*Carassius carassius*), pike (*Esox lucius*), and rudd *(Scardinius eurythrophtalmus*). This lake is a high predation environment and thus evaluated as a “replicate” to Lake Lauarvann.

In the area of the study populations, the Weichselian ice sheet likely retreated around 9–10 000 years before present [53]. However, there are signs of freshwater deposits from an inter-glacial period in this area [54,55]. We modelled the sea-level and ice-front position for this scenario using the Surfer 11.0 software (Golden Software; http://www.goldensoftware.com/products/surfer), where our results confirmed such a possibility for a pro-glacial lake being formed by an ice lobe damming the present-day northward draining of the Lauar area (Fig 1). A pro-glacial lake is a lake that is formed by a moraine (through damming actions) or by an ice dam during the retreat of a melting glacier. At the same time, the position of Lake Ulvenvann was still below the sea level and did not yet exist as freshwater lake (Fig 1).

### Samples

Samples used for life-history and morphological analyses were mostly based on the master thesis of Lien [57] using a subset of the material from Sandågrotta cave and Lake Lille Lauarvann. Parts of these data have earlier been published by Lien et al. [58]. In addition, a sample was collected from Lake Ulvenvann later on to supplement data (see description below). The current publication re-analyzes the original data in more depth and adds analyses on mtDNA (new samples from each of the populations for the purpose of mtDNA analyses).

### Life-history

Individuals were collected using plexiglass traps [59] and rod sieves for hand sampling. In the Sandågrotta cave, the traps were left out all year, and emptied once a month. In Lake Lille Lauarvann the traps were only left out during the ice-free season (May-October) and also emptied once a month. In this lake, rod sieves were used in addition to the traps since the traps yielded a very low catch, likely due to many predators (i.e. trout, perch, minnow, dragonfly larvae) often entering the traps. In addition, we sampled material from Lake Ulvenvann with plexiglass traps and rod sieves in September 1994, May 1995 and June 1995. Different parts of the original samples were used for different analyses as reported below.

**Fig 2:**
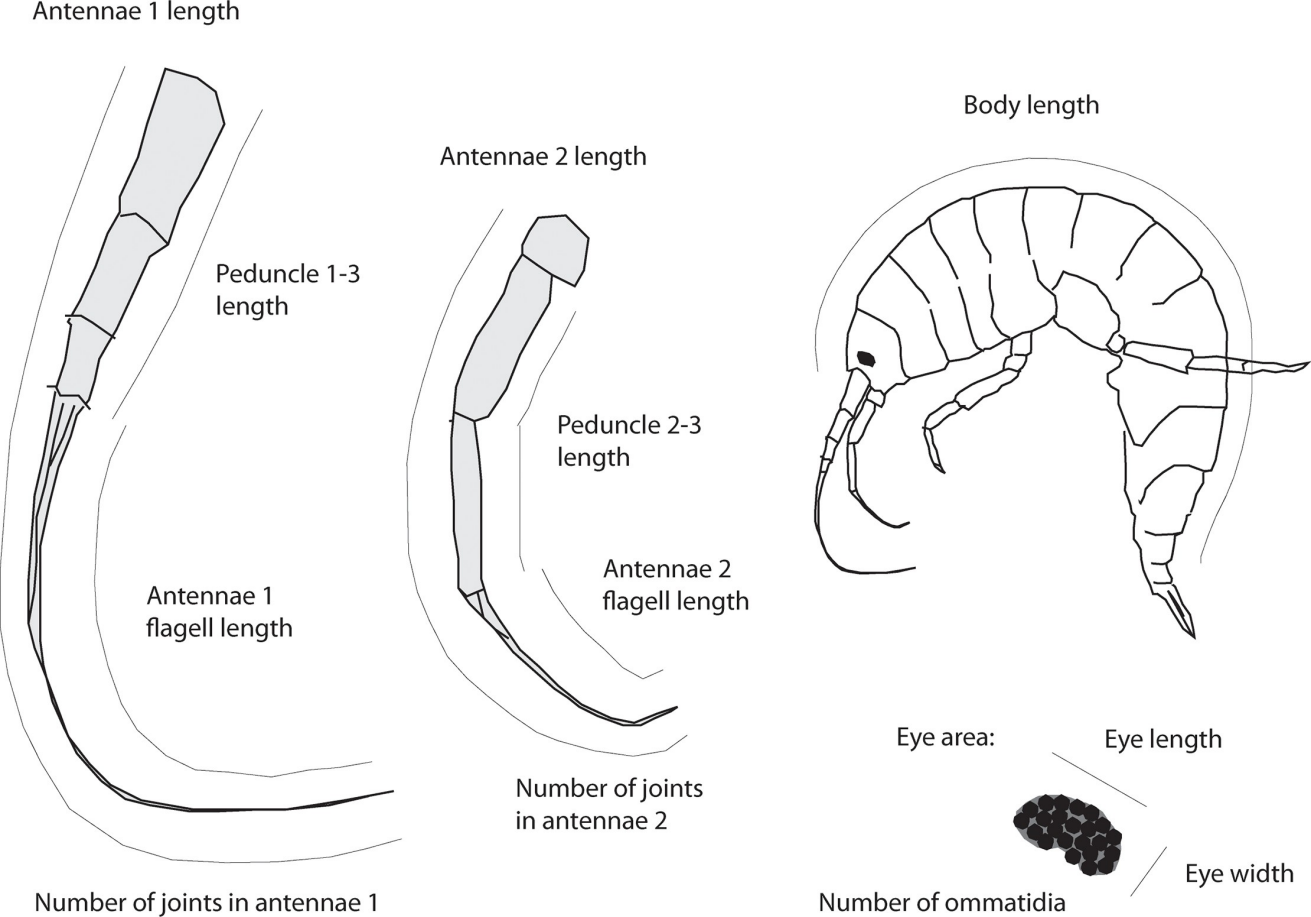
Morphological measurements conducted. The meristic and metric measurements scored for the three *G*. *lacustris* populations (note; only two legs have been depicted in this figure for ease of visualization).

The body length was measured along the lateral axis from the margin of the first antenna to the posterior end of third uropod in an extended individual (Fig 2). Here, we used a microscope (Zeiss; 10-40x). Determination of sex (males, females and juveniles, i.e. when no sex determination was possible) was conducted using the same microscope (Zeiss; 10-40x). The data for body length and sampling dates are given in S1 and S2 Tables. In all the statistical analyses conducted in this study we used the software JMP 5.0 [60].

In order to visually inspect if there are signs of more than one year class in the distribution of body lengths in the populations, we grouped existing material of immature individuals, males and females together and plotted body-length histograms partitioned into fall/spring samples.

We grouped sampling months into fall and spring samples to increase statistical power and biological inference due to few individuals sampled in some months testing if body length differed among the populations using ANCOVA with sex, season and location as variables.

The body length where 50% of the population reached sexual maturity was estimated for males and females separately across the three populations using a full-factorial design with population and body length as predictors and maturity stage (mature/non-mature) as the response variable in two separate nominal logistic regressions [61]. As few immature individuals were caught, we used the same immature individuals estimating body length at sexual maturity for both males and females. An initial test showed that sampling month or sampling season had no effect on the maturity probability (all tests P>0.05), thus individuals from different months/seasons were combined in a “yearly sample”. The data for maturity (i.e. defining males, females and immatures) are given in S3 and S4 Tables.

The number of eggs was counted in ovigerous females, where the length (L) and breadth (B) of five randomly chosen eggs were measured using a stereo-microscope (Wild Mz 8; 50x). The egg-volume (mm^3^) was estimated using a formula of an ellipsoid (volume = π x (4/3) x (L/2)^2^ x (B/2)) with means of the length and breadth values within individuals. To compare egg-volume we described egg-developmental stages on a scale from 1–6 following McCahon & Pascoe [62], but only used egg-stage 2-3-4 as they occurred in each population and as few other stages were seen. In addition, six individuals were removed from analyses as they had less than 12 eggs and deviated from body length x egg number association in their population. Most individuals were sampled in May-July 1995, and some in May 1996, however, sample-periods were combined into one time period to increase statistical power. An ANCOVA was performed using egg-number as the response variable and locality, body length, interaction locality x body length, and the egg-development stage as the predictor variables. Furthermore, an additional ANCOVA was run using egg-volume as the response variable and locality, body length, interaction locality x body length, egg-number, and egg-development stage as predictor variables. In both of these two analyses, the partial full-factorial strategy was implemented due to low sample sizes precluding a full-factorial design. The data for egg analyses (egg number and egg volume) is given in S5 Table.

### Morphometric and meristic traits

Due to the small sample size regarding populations x time periods, we compared the three locations using only two periods; September and October 1994 compared to May and June 1995). In order to get a demographically representative and statistical balanced sample of populations, we used a sample scheme to select specimens by location, collection date, sex, and body size. Here, specimens were first partitioned into size groups (small, medium, large) based on percentiles within each group of sex, location and sampling time. Four sexually mature specimens were then selected randomly within each combination of population, sampling time, sex and size. This sampling scheme comprised 12 specimens of each sex from each population and sampling time, 24 specimens from each population and sampling time, and ideally 48 specimens from each population. Thus, a total of 142 specimens were obtained and analyzed with 46 specimens from the Sandågrotta cave (fall 1994, N = 22; spring 1995, N = 24), 48 specimens from Lake Lille Lauarvann (fall 1994, N = 24; spring 1995, N = 24) and 48 specimens from Lake Ulvenvann (fall 1994, N = 24; spring 1995, N = 24). These samples were then used for all the morphological analyses (see below). The data for morphological analyses (i.e. 10 metric and meristic traits given below) is given in S6 Table.

Measurements were taken using photographs via a television screen (Assar Magnilink 21 color Monitor with BCZT camera), and a stereo-microscope (Leica Mz 6; 10-40x)(Fig 2). The meristic traits were counted using a stereo-microscope (10-40x). On the first antennae, we measured the total length (ANT1), and the sum of the three peduncle lengths (PEDS1). Flagellum length (FL1) was found by subtracting peduncle lengths from ANT1, where then number of flagellular articles (LEDD1) was counted. The total length of the second antennae (ANT2), the sum of the two outermost peduncles lengths (PEDS2) and flagellum length (FL2), which was found by subtracting peduncle lengths from ANT2, was measured. All the flagellular articles (LEDD2) were counted. The eye area (OAR) was estimated as the surface area of an ellipse with the major axis represented by the greatest dimension of the eye along the dorsoventral line and the minor axis represented by the shortest dimension of the eye perpendicular to the first axis. Numbers of ommatidia (OM) was counted in the right and left eye, where the mean was used in further analysis. Traits were log transformed before analyses.

We used general linear models for the set of analyses with location, season, sex, body length and interaction between location x body length, and between location x sex, as predictor variables. Here, we tested if the following variables were different in the cave than in the surface environments; (1) *Log* mean number of ommatidia (lnOM), (2) *Log* eye area (lnOAR) (mm^2^). We then tested if the following variables were different in the cave than in the surface environments; (3) *Log* total length of antennae 1 (lnANT1) (mm), (4) *Log* number of joints in antennae 1 (lnLEDD1), (5) *Log* sum of the three peduncle lengths in antennae 1 (lnPEDS1) (mm), (6) *Log* length of the flagellum in antennae 1 (lnFL1) (mm), (7) *Log* length of antennae 2 (lnANT2) (mm), (8) *Log* number of joints in antennae 2 (lnLEDD2), (9) *Log* sum of the three peduncle lengths in antennae 2 (lnPEDS2) (mm), and (10) *Log* length of flagellum in antennae 2 (lnFL2) (mm).

In order to reveal the multivariate divergence between the three populations we used all the morphological eye- and antennal traits analyzed together in a canonical discriminant analysis using population of origin as the grouping variable, pooling males and females. Here, we used the residuals for each of the *Log* traits from regressions on *Log* body length. A post hoc Tukey-Kramer test was used to test whether canonical axes differed among the environments.

#### Genetic analysis

In August 2010, we sampled 10 specimens, stored in 96% Ethanol, from each of the three populations of Sandågrotta Cave, Lake Lille Lauarvann and Lake Ulvenvann to amplify and sequence a portion of the cytochrome *c* oxidase subunit I gene (COI) mtDNA region to assess genetic relationships [25,63,64].

DNA extractions, PCR amplification using the universal Folmer et al. [65] COI primers, and DNA sequencing were performed as previously described [25]. In addition, we downloaded a set of 15 sequences from GenBank to evaluate the possibility of postglacial colonization of our Norwegian samples as compared to the available sequences from a much larger geographical area with representatives from North America (USA and Canada), Europe (Slovenia and Ukraine), Asia (China, Mongolia and Russia, Iran), and three genetically-related outgroup species, *G*. *lobifer*, *G*. *inberbus*, and *G*. *decorosus*. Maximum likelihood (ML), maximum parsimony (MP), and Bayesian inference methods, as implemented in PAUP v. 4b10 [66] and MrBayes v.3.2 [67], respectively, were employed to construct phylogenetic trees. Evolutionary model selection for ML and Bayesian analysis was conducted using jModeltest 2.1.4 [68]. Phylogenetic trees were rooted with the three outgroup species, which were genetically distinct from the remaining samples. For molecular dating we used two molecular clock calibration schemes, the first based on the interspecific divergence estimates of Hou et al. [69], and the second based on a crustacean-specific intraspecific rate of substitution of 5% sequence divergence per million years based on a wide cited study by Crandall et al. [70]. In the Hou et al. [69] study, species divergence times between *G*. *lacustris* and each of the following three species were obtained: *G*. *lobifer* (14.22 Ma), *G*. *inberbus* (12.36 Ma), and *G*. *decorosus* (4.16 Ma). The corresponding species divergence times based on the Crandall et al. [70] substitution rate combined with our observed average COI sequence divergences were *G*. *lobifer* (3.10 Ma), *G*. *inberbus* (3.30 Ma), and *G*. *decorosus* (2.89 Ma). We used those estimates as calibration points in our analysis, which was conducted using the Bayesian method implemented in BEAST 1.10.1 [71] employing the following models: strict molecular clock, a coalescent (intraspecific) prior, HKY+G nucleotide substitution, and a normal distribution with standard deviations from the Hou et al. [69] or Crandall et al. [70] as priors on the calibration nodes. The MCMC was run for 10 million generations sampled at 1000-generation intervals. Node ages, along with their 95% highest posterior density distributions, were obtained from the maximum clade credibility Bayesian tree constructed from the last 8 million generations of the MCMC run.

## Results

### Life history

**Fig 3:**
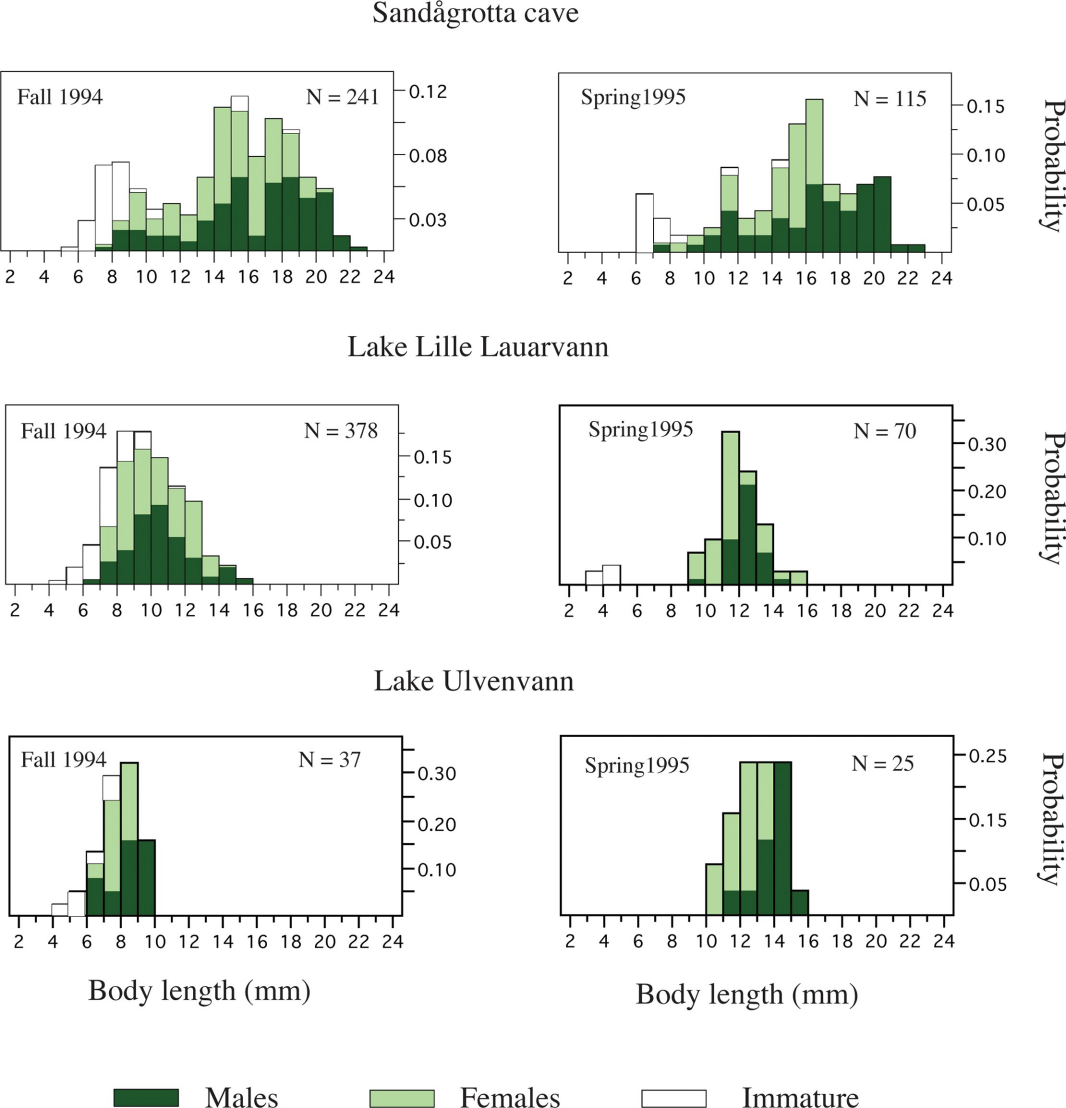
Body size distribution pattern. Autumn and spring body size distribution in the Sandågrotta Cave, and the surface populations of Lake Lille Lauarvann and Lake Ulvenvann. The color codes are as follows; white bars = immature individuals, ligth green bars = females and dark green = males. The vertical scale denotes the probability of finding different size-classes within these population. The sample size for each site and temporal comparison is given within each of the six figures.

The body-length distributions in the Sandågrotta Cave, Lake Lille Lauarvann and Lake Ulvenvann appeared different when we performed a visual evaluation of fall and spring samples of sexually mature males, females and immature individuals (Fig 3). The distribution of body lengths in the Sandågrotta Cave appeared as a multimodal distribution while it seemed roughly normal for Lake Lille Lauarvann and Lake Ulvenvann, revealing larger animals in the cave population. Thus, based on the body length distributions, we interpret that surface populations have a shorter life-cycle than the longer life cycle in the Sandågrotta cave population. Males seem generally larger than females in all three populations. In the ANOVA analysis, the body length differed significantly between the three sampling populations being affected by season (fall or spring samples) and sex (Table 1). Here, body length was larger in the Sandågrotta cave than in the two surface populations, males were larger than females, and spring samples were larger than fall samples (using additional LSM post-hoc comparisons). The maximum size in the Sandågrotta cave was 23 mm, while only 16 mm in both of Lake Lille Lauarvann and Lake Ulvenvann (Fig 3). Mean and variance estimates for body length is given in S7 Table, while body length boxplot-quantiles is given in S1 Fig.

**Table 1:** The results from test of body length differences between sampling locations.

Source	Response variable	N	R^2^	F-ratio	P-value
Model	Body length	866	0.58	240.34	<0.001
Location				314.16	<0.001
Season (Fall, Spring)				24.32	<0.001
Sex				217.10	<0.001

**Fig 4:**
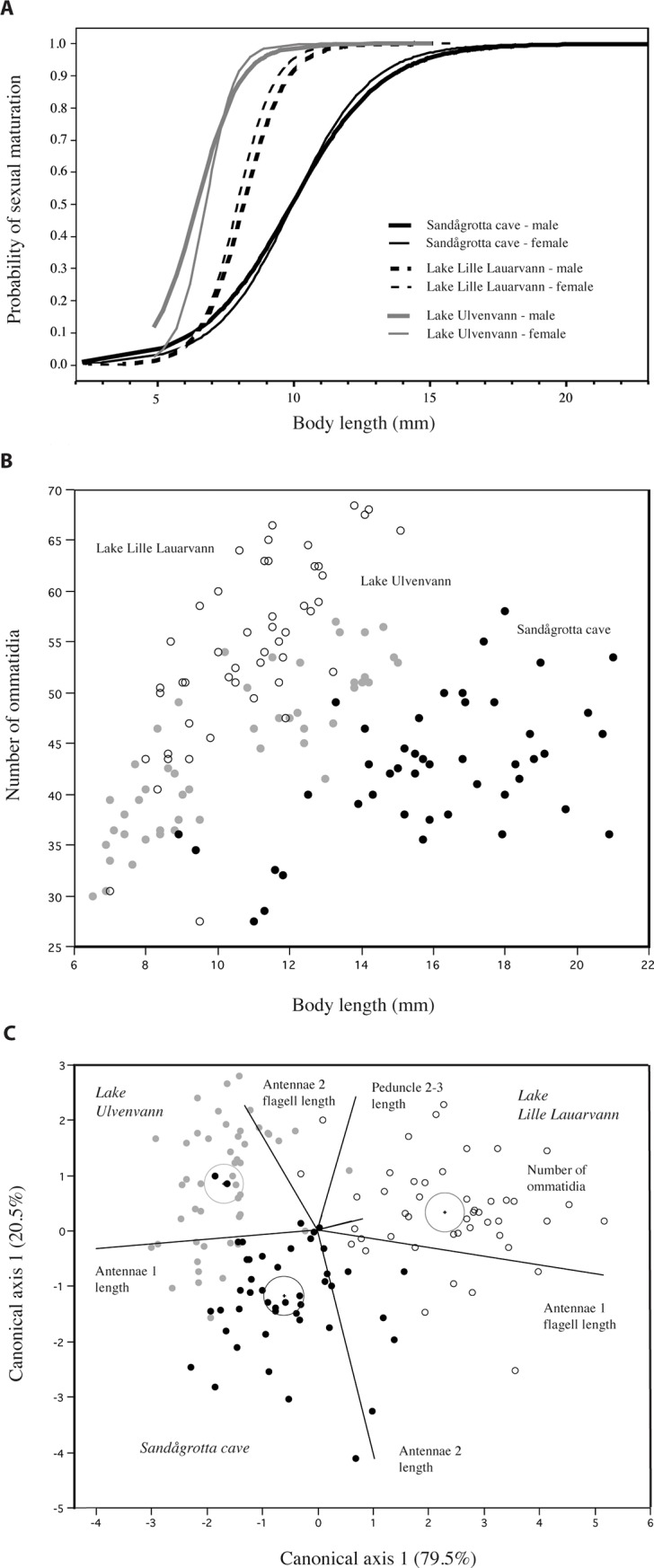
Maturation probability, ommatidia and canonical discriminant analysis. (A) Sexual maturation probabilities as a function of body size in the three populations estimated where 50% of the populations are sexually mature for males and females separately. (B) The relationship between body length and number of ommatidia in the Sandågrotta cave (black symbols) and in the two surface populations of Lake Lille Lauarvann (white symbols) and Lake Ulvenvann (grey symbols). (C) Canonical discriminant analysis results with vector importance (of traits) and where circle reveal 95% confidence ellipses of population means.

When estimating length at 50% sexual maturity in males and females separately the results showed interaction between populations and body length where maturation trajectories had significantly different slopes (Table 2, Fig 4A). Here, it was evident that males were maturing at a larger size in the Sandågrotta cave (9.9 mm) than in Lake Lille Lauarvann (8.2 mm) and Lake Ulvenvann (6.5 mm). The very same pattern emerged when comparing females across sites where Sandågrotta cave (10.0 mm) females had larger size at maturity than in Lake Lille Lauarvann (7.9 mm) and Lake Ulvenvann (6.8 mm). The maturation probability results are given in S3 Table.

**Table 2:** Length at sexual maturity for males and females in sampling locations.

Source	Sex	N	Df	χ2	P-value
Model	Males	498	5	314.33	<0.001
Location			2	31.65	<0.001
Body length (mm)			1	32.07	<0.001
Location x Body length (mm)			2	13.88	0.001
Model	Females	508	5	311.26	<0.001
Location			2	37.28	<0.001
Body length (mm)			1	20.51	<0.001
Location x Body length (mm)			2	12.79	0.002

The fecundity (i.e. the number of eggs) differed significantly (using additional LSM post-hoc comparisons) between surface and cave locations when scaled by body length where Lake Ulvenvann (LSM = 44.0 eggs) and Lake Lille Lauarvann (LSM = 45.8 eggs) did not differ from each other, while differing from Sandågrotta cave (LSM = 25.5 eggs) (Table 3). Only one other parameter, body length, was significant showing a positive relationship within populations between body length and fecundity. The association between body length and fecundity is given in S2 Fig.

**Table 3:** Gonad investment in fecundity (i.e. number of eggs) and egg volume (mm^3^).

Source	Response variable	N	R^2^	F-ratio	P-value
Model	Fecundity (egg number)	67	0.66	16.70	<0.001
Location				7.24	0.002
Body length (mm)				71.88	<0.001
Location x Body length (mm)				1.80	0.174
Egg development stage				2.72	0.074
Model	Egg size (egg volume, mm^3^)	67	0.90	69.90	<0.001
Location				12.15	<0.001
Body length (mm)				1.52	0.223
Location x Body length (mm)				2.66	0.078
Fecundity (egg number)				3.72	0.059
Egg development stage				26.54	<0.001

Egg volume differed significantly between the Sandågrotta cave and the two surface populations (which did not differ using LSM post-hoc comparisons), where Sandågrotta cave (LSM = 0.40 mm^2^) had larger eggs than Lake Lille Lauarvann (LSM = 0.23 mm^2^) and Lake Ulvenvann (LSM = 0.24 mm^2^) (Table 3). Egg volume also increased significantly with an increase in the egg developmental stage 2–4. The association between body length and egg volume is given in S2 Fig.

### Morphology

For all the ten studied morphological response variables, mean and variance values are given in the S8 Table, while boxplot-quantiles are given in S3 Fig.

#### Eye traits

The eyes of the individuals from the two surface lakes were kidney shaped, while they varied from kidney shaped or approximately triangular to more spherical in cave living individuals.

The mean number of ommatidia (variable: *Log* mean number of ommatidia, lnOM) differed significantly between the two environments (Table 4, Fig 4B) where Sandågrotta cave had fewer ommatidia than Lake Lille Lauarvann or Lake Ulvenvann. All populations were significantly different (LS means contrast P<0.001). Season had a small but significant effect where spring individuals had fewer ommatidia than fall individuals when compared across populations. The body length had a strong and significant effect on the number of ommatidia.

**Table 4:** Results for morphometric trait analyses.

Test	Source	df	*F*	*P*
*Log* mean number of ommatidia (lnOM)	Location	2	77.82	<0.001
R^2^ = 0.68	Season	1	4.77	0.031
N = 142	Sex	1	0.52	0.473
P < 0.001	Log body length	1	131.85	<0.001
	Location x Log body length	2	3.01	0.053
	Location x Sex	2	0.73	0.482
	Error	132		
*Log* eye area (lnOAR) (mm^2^)	Location	2	116.59	<0.001
R^2^ = 0.89	Season	1	12.33	<0.001
N = 142	Sex	1	21.97	<0.001
P < 0.001	Log body length	1	688.54	<0.001
	Location x Log body length	2	2.24	0.110
	Location x Sex	2	1.13	0.327
	Error	132		
*Log* total length of antennae 1 (lnANT1) (mm)	Location	2	51.88	<0.001
R^2^ = 0.95	Season	1	5.36	0.022
N = 142	Sex	1	22.60	<0.001
P < 0.001	Log body length	1	545.88	<0.001
	Location x Log body length	2	1.45	0.239
	Location x Sex	2	0.93	0.400
	Error	132		
*Log* number of joints in antennae 1 (lnLEDD1)	Location	2	5.38	0.006
R^2^ = 0.82	Season	1	10.72	0.001
N = 142	Sex	1	5.27	0.023
P < 0.001	Log body length	1	200.10	<0.001
	Location x Log body length	2	1.35	0.263
	Location x Sex	2	0.68	0.507
	Error	132		
*Log* sum of the three peduncle lengths in antennae 1 (lnPEDS1) (mm)	Location	2	0.31	0.732
R^2^ = 0.94	Season	1	3.00	0.086
N = 142	Sex	1	49.27	<0.001
P < 0.001	Log body length	1	716.96	<0.001
	Location x Log body length	2	1.17	0.313
	Location x Sex	2	3.85	0.024
	Error	132		
*Log* length of flagell in antennae 1 (lnFL1) (mm)	Location	2	60.64	<0.001
R^2^ = 0.93	Season	1	4.10	0.045
N = 142	Sex	1	11.63	<0.005
P < 0.001	Log body length	1	333.37	<0.001
	Location x Log body length	2	2.00	0.139
	Location x Sex	2	0.359	0.699
	Error	132		
*Log* length of antennae 2 (lnANT2) (mm)	Location	2	9.55	<0.005
R^2^ = 0.96	Season	1	3.44	0.066
N = 142	Sex	1	205.90	<0.001
P < 0.001	Log body length	1	884.14	<0.001
	Location x Log body length	2	3.42	0.035
	Location x Sex	2	0.23	0.795
	Error	132		
*Log* number of joints in antennae 2 (lnLEDD2)	Location	2	9.53	<0.005
R^2^ = 0.85	Season	1	1.49	0.224
N = 142	Sex	1	98.14	<0.001
P < 0.001	Log body length	1	145.63	<0.001
	Location x Log body length	2	2.55	0.082
	Location x Sex	2	1.90	0.154
	Error	132		
*Log* sum of the two peduncle lengths in antennae 2 (lnPEDS2) (mm)	Location	2	50.96	<0.001
R^2^ = 0.96	Season	1	4.82	0.030
N = 141	Sex	1	241.05	<0.001
P < 0.001	Log body length	1	829.89	<0.001
	Location x Log body length	2	0.25	0.078
	Location x Sex	2	0.59	0.554
	Error	132		
*Log* length of flagell in antennae 2 (lnFL2) (mm)	Location	2	5.73	0.004
R^2^ = 0.92	Season	1	0.53	0.467
N = 141	Sex	1	73.57	<0.001
P < 0.001	Log body length	1	401.06	<0.001
	Location x Log body length	2	6.45	0.002
	Location x Sex	2	0.09	0.914
	Error	132		

The area of the eye (variable: *Log* eye area in mm^2^, lnOAR) differed significantly between the two environments (Table 4), where Sandågrotta cave had a smaller eye area than did Lake Lille Lauarvann and Lake Ulvenvann. Again, all the three populations were significantly different (LS means contrast P<0.001). Moreover, the individuals from the spring sample had a significantly smaller eye area than the fall sample individuals when compared across all the three populations. Moreover, the males had a significantly smaller eye area than did females. A strong significant positive association was observed between body length and eye area.

#### First antennae traits

The length of the first antennae (variable: *Log* total length of antennae 1 (lnANT1) (mm) differed significantly among environments (Table 4) where Sandågrotta cave had longer antennae than Lake Lille Lauarvann and Lake Ulvenvann. All populations were significantly different (LS means contrast P<0.001). Season had a small significant effect showing that the spring sample individuals had smaller antennae than the fall sample individuals when compared across all three populations. Males also had a significantly larger antenna than did females. Body length was strongly significantly associated with the length of the antenna.

When excluding the first three basal peduncles in the first antenna, the number of joints in the first antenna (variable: *Log* number of joints in antennae 1 (lnLEDD1)) differed significantly among populations (Table 4) where Sandågrotta cave and Lake Lille Lauarvann had similar values, being significantly different (LS means contrast P<0.001) from Lake Ulvenvann. Season had a small significant effect showing that spring sample individuals had fewer joints in the first antenna than fall sample individuals when compared across all three populations. Males had significantly more joints in the first antenna than females. Moreover, body length was strongly and significantly associated to the number of joints in the first antenna.

The summed length of the three peduncles (variable: *Log* sum of the three peduncle lengths in antennae 1 (lnPEDS1) (mm) in the first antenna did not differ among the environments (Table 4) (LS means contrast P = 0.50). Season had no significant effect, but body length showed a strong and positive association with the summed length of the three basal peduncles in the first antenna. Sex had a significant effect where males had larger peduncle lengths than females. It was a significant interaction between population and sex showing that the divergence in lengths between the two sexes was largest in Lake Ulvenvann, Sandågrotta cave and lowest in Lake Lille Lauarvann (evaluated from a visual inspection of interactions).

The summed length of the flagellum in the first antennae (excluding the summed length of the three peduncles (lnPEDS1) (variable: *Log* length of flagellum in antenna 1 (lnFL1) (mm)) differed significantly among environments (Table 4). Here, Sandågrotta cave had a larger flagellum length than Lake Lille Lauarvann or Lake Ulven, all population comparisons being significantly different (LS means contrast P<0.001). Season had a small and significant effect where spring individuals had a smaller antenna length than fall individuals. Males had a significantly larger antenna than females. Body length was strongly and significantly associated with antennae length.

#### Second antennae traits

The length of the second antenna (variable: *Log* length of antenna 2 (lnANT2) (mm)) differed significantly among environments (Table 4) where Sandågrotta cave had a longer antenna than Lake Lille Lauarvann and Lake Ulvenvann. All populations were significantly different (LS means contrast P<0.001). Males had a significantly larger antenna than females. Body length was strongly and significantly associated with the length of the antenna. There was a significant interaction between location and body length showing that Lake Ulvenvann had a slower rate of increase (using visual inspection of interactions).

The number of joints in the second antenna (variable: *Log* number of joints in antenna 2 (lnLEDD2)) also differed significantly among the environments (Table 4), where the Sandågrotta cave population had more joints than Lake Lille Lauarvann and Lake Ulvenvann, all the three populations being significantly different (LS means contrast P<0.001). Males had more joints in the antennae than females. Body length was strongly and significantly associated with the number of joints in the second antenna.

The summed length of the two peduncles in the second antenna (variable: *Log* sum of the two peduncle lengths in antenna 2 (lnPEDS2) (mm)) differed significantly among environments (Table 4), where Lake Lille Lauarvann were longer than Sandågrotta cave and Lake Ulvenvann (LS means contrast P<0.001). Season had a weak significant effect where spring individuals had significantly a smaller peduncle length than fall individuals. Males had significantly larger lengths than females. Body length was strongly and significantly associated with the summed length of the two peduncles.

The length of the flagellum in the second antenna (variable: *Log* length of flagellum in antenna 2 (lnFL2) (mm)) differed significantly between the environments (Table 4) where Sandågrotta cave and Lake Ulvenvann were significantly larger than Lake Lille Lauarvann (LS means contrast P<0.001). Males had significantly larger flagellum than females. Body length was strongly and significantly associated with the length of the flagellum. There was a significant interaction between location x body length, showing that the steepest increase was seen in Sandågrotta cave (using visual inspection of interactions).

#### Multivariate phenotypic population differentiation

In the canonical discriminant analysis pooling sex only 16 out 142 individuals analyzed were misclassified to the wrong sampling population (11.4%), showing a high discriminant power (Wilks´ Lamda = 0.15, P<0.001) (Fig 4C). Here, the correct assignments for cave animals were 41/46 individuals to the Sandågrotta cave, miss-assignment of 1/46 to Lake Lille Lauarvann, and 4/46 to Lake Ulvenvann. Similarly, the correct assignment for Lake Lille Lauarvann animals were 43/47 to Lake Lille Lauarvann, miss-assignment of 2/47 to Sandågrotta cave and 2/47 to Lake Ulvenvann. Finally, the correct assignment for Lake Ulvenvann animals were 41/48 to Lake Ulvenvann, miss-assignment of 1/48 to Lake Lille Lauarvann, and 6/48 to Sandågrotta cave. The two canonical axes revealed 79.5% (CA1: eigenvalue = 2.93) and 20.5% (CA2: eigenvalue = 0.75) of the variation, respectively. Here, all the three populations differed significantly along canonical axis 1 (post hoc Tukey-Kramer test, P<0.05), where Sandågrotta cave and Lake Ulvenvann were more similar morphologically to each other than to Lake Lille Lauarvann. Along canonical axis 2, all populations also differed significantly from each other (post hoc Tukey-Kramer test, P<0.05), however, the two surface populations were most morphologically similar. The six most important biplot rays (i.e. directions of variables in the canonical space) are given in Fig 4C. These traits were the lengths of first and second antenna, first and second antennae flagellum lengths, the peduncle length in second antenna and number of ommatidia.

### MtDNA phylogeny and divergence times

In total, 15 COI sequences were analyzed; 10 obtained from each of the Sandågrotta Cave and Lake Lille Lauarvann populations, and five COI sequences from the 10 Lake Ulvenvann samples due to low DNA quality. A total of 12 unique haplotypes were present in these three populations (sequences are deposited in GenBank with the accession numbers: KJ831277-88, Table 5). The Sandågrotta Cave population had five haplotypes (*h1*-*h5*), Lake Lille Lauarvann five haplotypes (*h6*-*h10*), and Lake Ulvenvann only two haplotypes (*h11*-*h12*). Within the three Norwegian populations mean nucleotide diversity (π) levels were similar in the Lake Lille Lauarvann (0.0012) and Sandågrotta Cave (0.0015) populations, and roughly fourfold higher in the Lake Ulvenvann population (0.0055). Haplotype diversity (*H*_*d*_) was very similar among the three populations and varied between 0.40–0.50. There were between four to six polymorphic sites segregating within each population. For the phylogenetic analyses an additional 15 *G*. *lacustris* sequences were retrieved from GenBank (see Table 5).

**Table 5:** The *Gammarus lacustris* sequences used in comparative phylogenetic analysis. H denote haplotypes.**–**denotes missing information. Norwegian sequences are deposited in GenBank (with accession numbers KJ831277-88, while remaining sequences are retrieved from GenBank). Outgroup sequences: *Gammarus decorosus* (GenBank Acc. Number: JF965875), *G*. *lobifer* (JF965920), and *G*. *inerbus* (JF965902).

Population and individual number	Locality	Country	Longitude	Latitude	H	GenBank
Sandågrotta cave 2	Kongsberg	Norway	9.38	59.32	1	KJ831277
Sandågrotta cave 5, 6, 10–12, 19	Kongsberg	Norway	9.38	59.32	2	KJ831278
Sandågrotta cave 7	Kongsberg	Norway	9.38	59.32	3	KJ831279
Sandågrotta cave 13	Kongsberg	Norway	9.38	59.32	4	KJ831280
Sandågrotta cave 17	Kongsberg	Norway	9.38	59.32	5	KJ831281
Lake Lille Lauarvann 1–4, 9, 11	Kongsberg	Norway	9.38	59.33	6	KJ831282
Lake Lille Lauarvann 6	Kongsberg	Norway	9.38	59.33	7	KJ831283
Lake Lille Lauarvann 7	Kongsberg	Norway	9.38	59.33	8	KJ831284
Lake Lille Lauarvann 8	Kongsberg	Norway	9.38	59.33	9	KJ831285
Lake Lille Lauarvann 10	Kongsberg	Norway	9.38	59.33	10	KJ831286
Lake Ulvenvann 6, 11, 12	Asker	Norway	10.21	59.48	11	KJ831287
Lake Ulvenvann 9, 10	Asker	Norway	10.21	59.48	12	KJ831288
Voucher “SLOCHN001”	Bled	Slovenia	46.35	14.12	13	JF965915
Olkhon Island	L. Baikal	Russia	53.15	107.38	14	AY926671
Voucher “GLAC1”	L. Baikal	Russia	-	-	15	FJ756329
Neour pond	Ardebil	Iran	38.00	48.55	16	HQ198593
Selenge River	B. aimag	Mongolia	49.38	102.65	17	JF965917
Yanqing	Beijing	China	40.40	115.90	18	EF570315
Hoh Xil	Qinghai	China	35.43	93.60	19	EF570320
Haixi	Qinghai	China	37.30	97.20	20	EF570323
Voucher “IZCASIA0696”	Xinjiang	China	43.90	88.10	21	JF965916
Altun	Xinjiang	China	38.20	90.30	22	EF570321
Xainza	Tibet	China	30.90	88.60	23	EF570322
Voucher “09PROBE-008634”	Manitoba	Canada	54.76	93.95	24	HM425346
Voucher “MaGam000”	-	Canada	-	-	25	DQ889100
Blue Pond	Manitoba	Canada	53.41	98.52	26	AY529052
Yellowstone Lake	Wyoming	USA	-	-	27	GU066811
Ukraine	Liubliaz	Ukraine	25.47	51.85	28	JX899356

**Fig 5:**
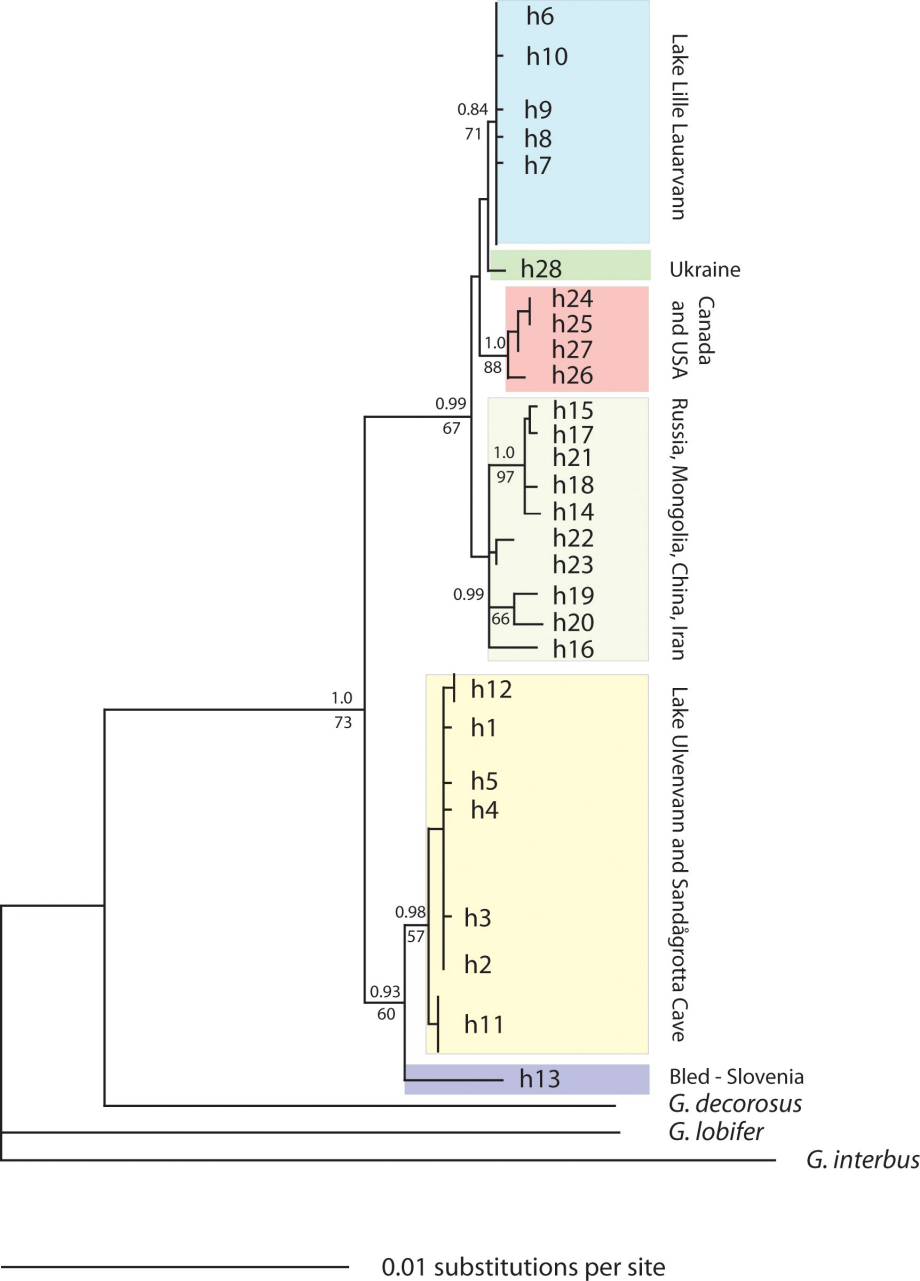
Consensus mtDNA-COI gene tree with bootstrap support provided. Maximum likelihood bootstrap (above) and Bayesian support (below)). The location codes are as follows: LL = Lake Lille Lauarvann, SC = Sandågrotta Cave, and LU = Lake Ulvenvann.

ML and Bayesian analyses gave the same optimal tree (Fig 5), which supported monophyly of the Lake Lille Lauarvann samples with moderate bootstrap (71%) and Bayesian clade credibility values (0.84), as well as the monophyly of a clade consisting of both the Lake Ulvenvann and Sandågrotta cave samples (57% bootstrap support, 0.98 clade credibility). Interestingly, despite their relative geographical proximity, European sequences from Ukraine and Slovenia obtained from GenBank clustered as sister groups to either the Lake Lille Lauarvann sequences (Ukraine), or to the Lake Ulvenvann/Sandågrotta cave clade (Slovenia). The monophyly of the latter clade was supported by ML bootstrap analysis (60%) and strongly supported by Bayesian clade credibility (0.93). Also of interest is the finding that these European clades did not cluster as sister groups. Rather, the Lake Lille Lauarvann clade clustered with the Asian and North American clades with moderate (67%) bootstrap support and strong Bayesian clade credibility (0.99). The Lake Ulvenvann/ Sandågrotta Cave/Slovenia clade was clearly distinct from remaining *G*. *lacustris* samples.

Mean divergence times based on the two calibration schemes were in general agreement (Table 6). The mean divergence time of *G*. *lacustris*, which was calculated from the mean of all the *G*. *lacustris* sequences including those outside Norway, was estimated at ~3.5 Ma using both calibration assignments, and these estimates were almost identical to those calculated for the exclusively Norwegian samples, which ranged between 3.39 and 3.42 Ma. Amongst the Norwegian populations, the Lake Lille Lauarvann, Lake Ulvenvann, and Sandågrotta Cave populations were estimated to be of the same approximate age (~ 0.2–0.6 Ma). The divergence times between the three Norwegian populations were less than the divergence time for *G*. *lacustris* as a whole. The divergence time between Sandågrotta Cave and Lake Ulvenvann was estimated as 0.25–0.79 Ma, and that between Sandågrotta Cave (or Lake Ulvenvann) and Lake Lille Lauarvann was significantly greater at 1.88–1.90 Ma. We note that since these divergence times are all derived from analysis of a single marker (COI), all are likely to be underestimates of the true divergence dates [72].

**Table 6:** Mean Bayesian divergence time (Ma) estimates using two different calibration schemes. The first scheme is based on calibration points derived from Hou et al. [69]. The second scheme is based on a widely used crustacean intraspecific substitution rate of 5% per million years from Crandall et al. [70]. The lower and upper 5% extreme posterior density values are shown in square brackets.

	Interspecific Calibration; Hou et al. [69]	Intraspecific Calibration; Crandall et al. [70]
Species Calibration Point (Fixed)		
*G*. *lobifer vs*. *G*. *lacustris*	14.22 [11.75, 16.69]	3.10 [2.63, 3.14]
*G*. *inberbus vs*. *G*. *lacustris*	12.36 [10.30, 14.42]	3.30 [2.94, 3.35]
*G*. *decorosus vs*. *G*. *lacustris*	4.16 [2.52, 5.81]	2.89 [2.33, 2.93]
Population Divergence (Estimated)		
Lake Lille Lauarvann	0.26 [0.08, 0.48]	0.20 [0.08, 0.33]
Lake Ulvenvann	0.19 [0.07, 0.34]	0.57 [0.22, 0.98]
Sandågrotta Cave	0.28 [0.12, 0.46]	0.31 [0.13, 0.54]
Sandågrotta Cave vs. Lake Ulvenvann	0.25 [0.11, 0.40]	0.79 [0.21, 1.40]
Sandågrotta Cave vs. Lake Lille Lauarvann	1.90 [1.12, 2.78]	1.88 [1.30, 2.54]
Lake Ulvenvann vs. Lake Lille Lauarvann*	1.90 [1.12, 2.78]	1.88 [1.30, 2.54]
Norway (LL/LU/SC)	3.42 [2.20, 4.81]	3.39 [2.40, 4.47]
*G*. *lacustris*	3.45 [2.23, 4.83]	3.46 [2.46, 4.52]

*The divergence time between Lake Ulvenvann and Lake Lille Lauarvann is the same as the divergence time between Lake Ulvenvann and Lake Lille Lauarvann because, taken together, the Lake Ulvenvann and Sandågrotta cave populations are a monophyletic group.

## Discussion

According to our hypotheses that life-history parameters and phenotypic traits were similar in the cave and surface populations, the results suggest that most of the hypotheses should be rejected. The results suggest that cave and surface environments harbor populations with different life-history and phenotypic traits. First, the life-history cycle in the cave appeared longer with delayed sexual maturation in contrast to surface populations with a shorter life-cycle and early sexual maturation. Secondly, the gonad allocation appeared altered in the cave environment resulting in lower fecundity and larger eggs than in surface populations. Further, the antennae (first and second antennae) were larger in the cave than in the surface populations. Finally, eye traits (number of ommatidia and eye-area) in the cave were reduced compared to the surface populations who each had larger eyes and more ommatidia. These phenotypic patterns emerged when traits were scaled by body size. As the Sandågrotta cave to our knowledge is the only “known” permanent cave-living population of *G*. *lacustris* worldwide we should merit this system special protection both nationally and internationally. In the following we discuss mechanisms behind our finds and compare results with similar pairs of surface—cave dwelling populations in a geographically remote and phylogenetically independent habitat specialization in North American *G*. *minus* evaluating if trait divergences and convergences to cave—surface environments are replicated in two genetic sister clades.

### Post-glacial colonization scenarios

The post-glacial colonization history of *G*. *lacustris* in Northern Europe based on allozyme data [72] suggests that a northeastern and a southwestern genetic lineage exist in Norway. Here, *G*. *lacustris* could have colonized southern parts of Norway through open waterways from ice-age refugia in the east from Russia/Siberia through the huge and temporally limited ice-age lakes from Russia [73] westwards to Sweden and Norway. The second colonization route seems to have occurred from the south from glacial refugia close to central Europe and the European Alp region, then up to Denmark and across to Sweden and Norway. The dispersal of *G*. *lacustris* has likely been via watersystems where high water-falls were barriers for upriver migration. It has been suggested that *G*. *lacustris* can spread by attaching to the feathers of water birds for up to 13 hours in the air [74], revealing a possibility that such transport avenues could partly explain the large number of *G*. *lacustris* in higher elevated lakes in Norway or Fennoscandia with no possibility for waterway colonization.

Looking at the retrieved COI-sequences in our study, we found two main clusters based on Bayesian credibility values. Here, the first main cluster comprised a sequence from Slovenia, with the Sandågrotta Cave and Lake Ulvenvann (a credibility value of 0.93). Slovenia was basal to the Sandågrotta Cave and Lake Ulvenvann clade (credibility value 0.98). The grouping of Lake Ulvenvann and the Sandågrotta Cave with Slovenia suggests that the two Norwegian populations were colonized from the southeast, in support of colonization scenarios suggested in Vainio & Väinölä [72] and Alther et al. [75]. This clade is already recognized as the Western and Central European clade by Vainio & Väinölä [72], and termed as a new species; *G*. *alpinus* by Alther et al. [75]. However, this species delineation was suggested to be wrong by Väinölä et al. [76], rather being a part of *G*. *lacustris* in the Western and Central European clade. The second main cluster (credibility value of 0.99) grouped Lake Lille Lauarvann with a sequence from Ukraine (credibility value of 0.84). This clade is also previously recognized by Vainio & Väinölä [72] and Alther et al. [75], being distributed across Skandinavia, Eurasia and North America according to Väinölä et al. [76]. We infer that the Lake Lille Lauarvann population likely originates from an ancestral population that arrived from an eastern and northern glacial refugia situated in Russia/Siberia.

With regard to the timing and putative colonization scenarios of the Norwegian populations we can discuss genetic patterns with regard to glacial geology. The first proposed scenario is that the Sandågrotta cave population arrived at the same time as, or a bit later than, the Lake Lille Lauarvann population approximately 10 000 years before present (ybp). This scenario does not fit with the phylogeny or divergence time estimates we have obtained in this study. Although we find that the two populations were established at approximately the same time, 0.20–0.26 Ma (Lake Lille Lauarvann) or 0.28–0.31 Ma (Sandågrotta cave), the two populations were independently derived, and therefore do not share a most recent common ancestor. Thus, as there is no sign of shared haplotypes between the two populations (being reciprocally monophyletic), and given the estimated divergence time of ~1.9 Ma between the two populations, the first scenario seems rather unrealistic. An alternative scenario is that these two populations could have been colonized at the same time, but that the cave population lineage was outcompeted in the surface environment and that the surface population lineage was outcompeted in the cave environment. However, this scenario also seems unlikely as one would expect some successful mating leading to genetic exchange of mtDNA sequences, and consequently the presence of shared haplotypes between the two populations. A third alternative here is that we do not have power to resolve this hypothesis given the relatively small sample size of sequenced specimens from each population. The cave population has likely colonized the cave from downstream in the river, or groundwater system, as no lakes or water bodies currently exists upstream the cave system. It is not likely that the cave population could have migrated from Lake Lille Lauarvann either as there currently exists a 10 m high waterfall acting as an upward migration barrier. Segerstråle [73] suggested that *G*. *lacustris* could attach itself to duck feathers and being transported for several hours before being released in a new water location. However, it seems unlikely that *G*. *lacustris* colonized the cave attached to duck feathers being released close to the cave. This is also the case given the level of genetic variation observed in the cave population as compared to the two other surface populations which suggest a moderate founder population entering the cave. The Sandågrotta cave population clustered with the Lake Ulvenvann population, being reciprocally monophyletic to Lake Lille Lauarvann. Given the genetic difference between the Sandågrotta cave population and the Lake Ulvenvann population it seems likely that these two populations represent two temporal colonization events from putatively the same ancestral genetic lineage. Some support can be found using glacial geology where it is evident that Lake Ulvenvann did not originate before ca 4 000 ybp due to isostatic rebound. With regard to the cave populations, this could have been founded during one of the many interstadial periods during the last glacial-interglacial cycle. During these interstadials, larger parts of Norway were likely ice-free. This scenario assumes that the Lauar-area at that time had an available water body that could be colonized, a water body at a sufficient altitude allowing animals to enter the cave directly or through “flat slope” rivers for upward migration. If this was the case, the cave population survived in the cave during the remaining ice-age cold periods, while the remaining populations outside in the surface area went extinct due to the ice-cover. Some support for a putative pro-glacial temporal lake are supported in the glacial geology, where undisturbed glacial clay deposits have been found even at elevations 100 meter higher than the current postglacial marine limit [54,55]. These deposits were made before the Weichsel III (Marine isotope stage (MIS) 2) glacial advance [77], which culminated ca 18 000–20 000 ybp. These deposits are assumed to be from an icefree period in the end of the last glaciation [54], but opens up the possibility that they can be deposited during the Middle Weichselian interstadial prior to the great Weichselian advance [55]. Thus, it seems reasonable to infer that during one of these interstadials some form of glacial deposits made it possible for a temporal lake to exist at the Lauar plateu area that lined up with the cave entrance. Also, it seems likely that the cave existed before the last ice-age started, i.e. before 120 000 ybp. This claim is supported from analyses of a stalactite in a closely situated cave in the same water drainage at approximately the same altitude. Here, U/Th radiometric dating showed an age of at least 60 000 ybp [51]. Since the stalactite was formed after the cave dried up (as they only grow in air-filled passages, preferably during ice free periods), the cave system must be much older than this age, maybe from a warm period before the last ice age. The question has been raised as to whether animals could have survived the ice age(s) in caves. In the Castleguard Cave, Canada, mostly situated under an active ice cap, the amphipod *Stygobromus canadensis* has been found living inside the cave in ponds/groundwater under the ice. This species is only known from this cave [78] and could potentially have survived the ice age in that cave. Long term glacial refugium of genetic lineages in *Gammarus fossarum* closer to the ice sheets than earlier thought was observed by Copilas-Ciocianu et al. [79]. A study by McInerney et al. [80] implied long term resilience for millions of years to extreme climatic changes in *Niphargus* species in the British isles including the Pleistocene Ice age. Another example comes from Kornobis et al. [81] suggesting that two subterranean amphipod species (*Crangonyx* spp.) survived repeated glacial periods underneath the ice sheets during ice ages in Iceland. These studies could further lend support to other cases in other species–such as potentially in our *G*. *lacustris*.

### Comparison of *G*. *lacustris* and *G*. *minus*

In general, the life history and phenotypic trait changes observed in the Sandågrotta cave population as compared to the two lake surface populations seem commensurate with yet other studies comparing surface and cave dwelling animals [8,19]. Thus, a somehow predictable difference seem to exist between such divergent habitats, suggesting that a set of mechanisms may be important operators in life history and trait divergences. However, cave animals often lack extant members that are closely genetically related to comparable surface populations, barring a good contrast of divergent environments with regard to changes during colonization of caves. As such, the Norwegian *G*. *lacustris* system may represent a good model system to infer the early steps in life history and trait changes. An additional useful approach to evaluate if natural selection has been influential in driving trait changes in caves is to compare organisms on a phylogeny to see if the same changes occurs independently as a response to similar environments. Here, we compare the evolution of a pair of surface and cave dwelling populations in *G*. *lacustris* in Norway with an independent evolution of similar surface-cave pairs in its genetic sister species *G*. *minus* in North America. It is reported that *G*. *minus* has surface and cave dwelling populations where not all cave populations show strong shifts in life history and traits from surface populations to cave living populations [19].

In our *G*. *lacustris* cave-surface contrast we observed that the life history of the Sandågrotta cave population appeared more prolonged than in surface populations (based on body size distributions). Here, one interpretation could be that a one year cycle existed in the two surface populations while a two or three year cycle existed in the cave population. Alternatively, the cave population could have a multimodal body size distribution due to co-occurrence of several generations less impacted by seasonality than the surface populations. Such pattern with regard to surface-cave populations were also observed in *G*. *minus* when using head lengths as a proxy for body size [11,82–84]. The length at sexual maturity was larger in the Sandågrotta cave than in surface populations. We find no publications reporting such studies in *G*. *minus*. However, it seems likely to infer that the same pattern may be present in *G*. *minus* based on body size differences. In the Sandågrotta cave also the gonad investment was smaller than in the surface population with larger and fewer eggs in the cave environment when scaled by body size. Glazier [85] studied ten mid-Appalachian (USA) spring populations of *G*. *minus* and found that in presence of sculpins (*Cottus* spp) amphipods produced more and smaller eggs than in populations without sculpins. This reflects impact from predators on life history evolution and is relevant for our surface-cave contrast for *G*. *lacustris*, as no fish exists in our caves but are present in the two surface populations. With regard to phenotypic traits, we found reduced ommatidia number, eye area and larger length of first and second antenna in the Sandågrotta cave when scaled by body size. In *G*. *minus*, populations are often divergent with fewer ommatidia, smaller eye area and longer antennae in caves than in surface populations [19,23,84]. Glazier & Deptola [86] found that *Gammarus minus* had larger eyes in freshwater springs with several fish predators. In *G*. *minus* the variation in ommatidia and eye area varies among caves and spring populations as well as within the caves [11]. In both the Sandågrotta cave and in cave living *G*. *minus*, the body coloration is rather pale whitish compared with brown-green coloration in surface populations (own observations, not quantified) [19]. However, the body coloration in the Sandågrotta cave *G*. *lacustris* becomes more pronounced upon exposure to sunlight and surface related food items (based on own observations from keeping individuals in light and dark aquarium conditions for years). Such labile coloration seems also present in *G*. *minus* [19]. Thus, coloration may be due to availability of food and light induction / pigmentation.

In contrast to *G*. *minus*, where standing levels of genetic variation at the COI locus differed substantially among cave populations with low levels of variation and surface populations with high levels of variation [25], genetic variation in the Sandågrotta cave population of *G*. *lacustris* was not necessarily lower than that of the two surface populations. The Sandågrotta cave and Lake Lille Lauarvann populations exhibited nearly identical levels of variation (mean nucleotide diversity π = 0.0015 and 0.0012, respectively) that were roughly fourfold lower than in the Lake Ulvenvann population (π = 0.0055), which comprised two divergent haplotypes among the relatively small number of individuals sequenced. In terms of the overall magnitude of variation at the COI locus, levels of variation in the three Norwegian populations of *G*. *lacustris* (π = 0.0012–0.0055) were comparable to those observed in most of the 15 *G*. *minus* populations (π = 0.0000–0.0185) in Carlini et al. [25]. It is difficult to make conclusions on levels of genetic variation in Norwegian cave and surface populations.

Thus, it seems that the life-history and trait-similarities in pairs of surface and cave living populations of *G*. *lacustris* and *G*. *minus* may suggest action of similar mechanisms in two evolutionary independent systems, pointing towards predicted optimum in cave related shifts.

### Cave related trait shifts: Natural selection, genetic drift or phenotypic plasticity?

Our findings may suggest that the life-history and phenotypic traits could be adaptations towards living in a cave environment as contrasted to the surface environment. However, traits may also reflect phenotypic plasticity, such that habitat associated trait differences may simply be a function of the living environment through e.g. temperature-diet-predation conditions. With regard to the prolonged life-history in the Sandågrotta cave, this could simply result due to lower temperature where the metabolic rate is reduced at lower temperature in such that a longer time span is needed for the completion of the life-cycle. In the surface-cave contrast this fit well with the Q_10_ parameter, i.e. a 2–3 reduction in metabolic rate with 10°C reduction in temperature, where the cave environment had only half of the temperature sum of the surface environment. Also, the nutrient productivity is likely very different in surface and cave environments, where the cave environment may be nutrient depleted. Thus, both a colder environment and less food may result in a prolonged life cycle in the cave compared to surface environments. Several studies support this claim as a prolonged life-cycle has been observed in high-elevation and cold-water living *G*. *lacustris* populations [31,34,87]. Also, the length distribution of *G*. *lacustris* individuals is associated with the length of the life-cycle where larger animals are found in populations with prolonged life-cycles [31,43]. Optimal gonad investment may differ in surface and cave environments due to the joint influence of temperature, food and predation regime (less predation in caves). In a study along an elevation gradient, Wilhelm & Schindler [31] found that *G*. *lacustris* females at higher altitudes produced larger and fewer eggs, that larger eggs had longer incubation time, and that young from larger eggs were larger. Reproductive investment was found to be correlated with female body size in *G*. *minus* by Glazier [49,50]. Thus, life-history traits are integrated. The same mechanisms may also explain the observed shifts in life-history in *G*. *minus* cave populations compared to surface populations in North America.

In *G*. *lacustris*, the cave population had longer antennae compared to the two surface populations. This may be a result of the larger body size in the cave due to the prolonged life-cycle. A correlation between body length and the first and second antennae in each of the three populations showed that they were highly correlated (antennae 1: R^2^ between 0.78–0.86, P<0.001, antennae 2: R^2^ between 0.68–0.83, P<0.001; based on non-log transformed values). Except for the result that antennae 2 in Lake Ulvenvann had a slower slope with body length than the two other populations, remaining correlations had non-significant interaction effects between population and body length. Thus, an increase in body length is sufficient to explain differences in antennae length in the cave population compared to surface populations. However, when looking at the residuals of the log-body length vs log-antennae 1 length (sex pooled), the Sandågrotta cave population had a significantly larger residual value than Lake Lille Lauarvann and Lake Ulvenvann (R^2^ = 0.28, N = 142, P<0.001). The same analysis, but now using log- antennae 2 resulted in no significant difference (R^2^ = 0.03, N = 142, P<0.098). This result deviates from the analysis reported in Table 4, and may either suggest that the allometric scaling with body length of antenna 1 length differs between the three populations, and/or that antennae 1 length is under stronger positive selection for a larger length in the Sandågrotta cave than in the two surface populations. With regard to the observed reduction in ommatidia number and eye size, the distribution of body lengths in the three populations seems insufficient to explain the pattern in the Sandågrotta cave population. As such, the reduction of ommatidia and reduced eye size can be interpreted to be adaptations to the cave environment where energy allocated to non-adaptive structures could be selected against.

Support for our general findings on trait divergences inferring evolutionary mechanisms is found in Jones et al. [23] who detected a pattern of directional selection in *G*. *minus* males for smaller eyes in caves and larger eyes in surface populations using mating pairs versus non-mating individuals. Other studies have reported signatures of directional selection for increased body size and longer antennas in *G*. *minus* in cave populations compared to surface populations [88,89]. In *G*. *minus*, separate invasion of cave environments from surface populations in different drainages imply that evolution of cave traits are replicated and predictable, strongly suggesting that similar traits result from similar selection pressures [19]. Also, high heritability estimates for antennae and eye traits have been demonstrated for *G*. *minus* populations in cave and surface environments [20]. In *G*. *minus*, a consistent pattern of eye-antenna genetic correlations between populations in the same habitat (cave positive versus surface centered around 0) across drainage basins was found, potentially suggesting a negative pleiotropic link between the traits [20]. This suggests that either both of these traits are under directional selection, or that only one trait is under selection whereas the other trait is neutral or nearly neutral and change due to pleiotropy or physical linkage in the architecture of the genome. Thus, it seems plausible to assume that antennae and eye-traits in *G*. *lacustris* are heritable in our populations and could be influenced by directional selection.

However, genetic drift in populations may also result from small founder populations affecting changes in morphology in surface living ancestors and derived cave populations. Indeed, genetic studies have shown that cave living populations of various organisms often have low genetic diversity [26–29]. With regard to nucleotide diversity (π), the Sandågrotta cave (0.0015) had a lower value than Lake ulvenvann (0.0055). However, Lake Lille Lauarvann (0.0012) had roughly the same value as in the cave. Thus, our data is not sufficient to reach a general conclusion as we only have one cave population and two surface populations. Further, under a genetic drift-based scenario one should not expect a replicated pattern in trait shifts between surface and cave populations and species. In general, a replicate shift is observed when comparing surface and cave populations in diverse taxa [8]. In particular, it seems that apparent cave traits are replicated at least within *G*. *minus*, a pattern mirrored also in our Norwegian populations of *G*. *lacustris*. Thus, natural selection may potentially be a likely and important driver of both eye- and antennal traits in organisms in cave systems, where also random genetic drift may be influential with regard to the rate of adaptation and the level of genetic variance upon where natural selection may act. To evaluate if selection has acted on the cave life history and phenotypic traits one should ideally perform common garden experiments varying both temperature and food conditions.

### Conservation biology and management

To our knowledge the occurrence of *G*. *lacustris* in the Sandågrotta cave represents the only permanent living cave population of this species worldwide. It seems to be characterized by different life-history and morphological traits which separates this population from the related surface population. As such, it is reasonable to suggest that the cave-environment has set the frame for the changes in the traits observed in the Sandågrotta population. Given the rareness of permanent and obligate cave populations of animals in Norway in general, and the apparent cave-related traits in the Sandågrotta cave population, the *G*. *lacustris* population should merit special conservation status in management. It is very important that the cave-environment is conserved together with the *G*. *lacustris* population to protect it for the future. A set of challenges emerges when debating conservation priority of organisms and populations. Here, the overall goal of conservation biology should be to secure the genetic integrity and the evolutionary process, in a way that anthropogenic impacts will not constrain future evolutionary trajectories [90,91]. It is a concern that given limited resources in biodiversity conservation, the priority for protection should ideally be associated with evolutionary distinctness of the taxa considered [92,93]. However, most of the methods used for identification of distinctiveness are debated [94]. The development of "Evolutionary Significant Units" (ESU´s), and “Management Units (MU´s)” has surfaced as a response to this kind of complexities when attempting to prioritize among taxonomic levels when considering conservation units below the species level [95–98]. Here, ESU´s are often defined based on genetic surveys of mtDNA where populations are reciprocal monophyletic with regard to haplotypes. For MU´s, populations should display significant divergence of allele frequencies (or haplotype frequencies) at nuclear (or mitochondrial loci). Finally, it should be evaluated if a population represents an important part of the evolutionary legacy of the species. Such evaluations should ideally be based on neutral genetic markers, selection on the genome and behavioral, phenotypic and physiological adaptations. Frazer & Bernatchez [99] reviewed the ESU concept and gave a context-based framework for delineating ESU`s.

Based on the COI-mtDNA region it is evident that the Sandågrotta cave population and Lake Ulvenvann are highly differentiated from Lake Lille Lauarvann based on the high bootstrap values. Apparently, these populations share a more common evolutionary history compared to Lake Lille Lauarvann. This is also supported by the multivariate phenotypic analysis where Lake Ulvenvann are more similar to the Sandågrotta cave population than either are to the Lake Lille Lauarvann population. The Sandågrotta cave and Lake Ulvenvann are also differentiated in the COI-mtDNA haplotypes, but with lower bootstrap. Thus, all the three populations have reciprocal monophyletic haplotypes and could represent three ESU´s. However, we stress that we have a rather small sample size within populations and also a small sample size of compared populations in general when also using GenBank data. Thus, one should in general be careful with strong interpretations based on the small sample sizes.

Considering molecular divergence, it seems reasonable to infer that the three populations were isolated into at least two glacial refugia (one or two for the Sandågrotta cave population and Lake Ulvenvann) and one for Lake Lille Lauarvann, that secondarily met in a contact zone after colonization. With regard to the phenotypic and life-history differences among the three populations, it seems reasonable to suggest that these may partly represent adaptations (and/or potentially phenotypic plastic traits) that have developed in different environments. Given the rare occurrence of one permanent cave living population of *G*. *lacustris*, the Sandågrotta cave population represents an important part of the evolutionary legacy of this species. The management authorities in Norway (Norwegian Environment Agency) have used our results and have given the Sandågrota Cave special protection (Nature Reserve) as it now is closed for the public in the lower parts (two entrances where most of the *Gammarus lacustris* individuals live), while being open in the upper part (where no *G*. *lacustris* individuals are found). The cave population of *G*. *lacustris* is still vulnerable to chemical spills/garbage etc. from the upper entrance where people can enter. However, the management restriction of humans to access only the upper part of the cave has been traded off for valuable experience for humans in nature versus the specific protection of this endemic population of *G*. *lacustris*. This process has involved researchers, the landowner and management authorities and is an example of a successful implementation based on science.

## Supporting information

S1 Table Individual body length. (XLSX)

S2 Table Body length and sampling dates. (DOCX)

S3 Table Maturation of males and females. (XLSX)

S4 Table Time period data for maturation. (DOCX)

S5 Table Raw data egg number and volume. (XLSX)

S6 Table Raw data for morphological analyses. (XLS)

S7 Table Mean and variance of body length. (XLSX)

S8 Table Mean and variance of morphological traits. (XLSX)

S1 Fig Body length quantiles. (DOCX)

S2 Fig Egg volume and egg number. (DOCX)

S3 Fig Morphological traits quantiles. (DOCX)
