# Supplementary material for: Morphology and life history divergence in cave and surface populations of Gammarus lacustris (L.)
Source: PLoS One. 2018 Oct 25;13(10):e0205556. doi: 10.1371/journal.pone.0205556 (PMC6201897; doi:10.1371/journal.pone.0205556)
Supplement: S2 Table — (DOCX) [file pone.0205556.s002.docx]

**S2 Table. Body length and sampling dates.**

Data used for body length analyses (locality, sex, maturity stage, season, dates, N individuals).

| Locality | Stage | Season * | N summed |
| --- | --- | --- | --- |
| Sandågrotta cave | Males | Fall/Spring 1994/1995 (59) + Fall 1995 (42) | 101 |
|  |  | Spring/Summer 1995 (58) | 58 |
|  | Females | Fall/Spring 1994/1995 (55) + Fall 1995 (45) | 100 |
|  |  | Spring/Summer 1995 (44) | 44 |
|  | Immature | Fall/Spring 1994/1995 (12) + Fall 1995 (28) | 40 |
|  |  | Spring/Summer 1995 (13) | 13 |
|  |  |  |  |
| Lake Lille Lauarvann | Males | Fall/Spring 1994/1995 (64) + Fall 1995 (78) | 142 |
|  |  | Spring/Summer 1995 (29) | 29 |
|  | Females | Fall/Spring 1994/1995 (71) + Fall 1995 (91) | 162 |
|  |  | Spring/Summer 1995 (36) | 36 |
|  | Immature | Fall/Spring 1994/1995 (16) + Fall 1995 (58) | 74 |
|  |  | Spring/Summer 1995 (5) | 5 |
|  |  |  |  |
| Lake Ulvenvann | Males | Fall/Spring 1994/1995 (17) | 17 |
|  |  | Spring/Summer 1995 (12) | 12 |
|  | Females | Fall/Spring 1994/1995 (14) | 14 |
|  |  | Spring/Summer 1995 (13) | 13 |
|  | Immature | Fall/Spring 1994/1995 (6) | 6 |

* Explanation for season (with different sampling dates):

- Sandågrotta cave: Fall/Spring 1994/1995 (09.10.94, 11.11.94, 14.12.94, 03.02.95, 03.03.95, 31.03.95), Spring/Summer 1995 (26.06.95, 19.07.95), Fall 1995 (17.08.95).
- Lake Lille Lauarvann: Fall/Spring 1994/1995 (30.09.94, 09.10.94, 11.11.94), Spring/Summer 1995 (26.05.95, 26.06.95, 19.07.95), Fall 1995 (17.08.95, 17.09.95, 05.10.95).
- Lake Ulvenvann: Fall/Spring 1994/1995 (01.09.94), Spring/Summer 1995 (10.05.95, 15.06.95).
