# Supplementary material for: Morphology and life history divergence in cave and surface populations of Gammarus lacustris (L.)
Source: PLoS One. 2018 Oct 25;13(10):e0205556. doi: 10.1371/journal.pone.0205556 (PMC6201897; doi:10.1371/journal.pone.0205556)
Supplement: S4 Table — (DOCX) [file pone.0205556.s004.docx]

**S4 Table. Time period data for maturation.**

Data for length at sexual maturity analyses (analyses, locality, maturity stage, season, dates, N individuals).

| Analyses | Locality | Stage | Season * | N summed |
| --- | --- | --- | --- | --- |
| Males | Sandågrotta cave | Mature | Fall/Spring 1994/1995 (59) + Spring/Summer 1995 (58) + Fall 1995 (42) | 159 |
|  |  | Immature | Fall/Spring 1994/1995 (12) + Spring/Summer 1995 (13) + Fall 1995 (28) | 53 |
|  |  |  |  |  |
|  | Lake Lille Lauarvann | Mature | Fall/Spring 1994/1995 (64) + Spring/Summer 1995 (29) + Fall 1995 (78) | 171 |
|  |  | Immature | Fall/Spring 1994/1995 (16) + Spring/Summer 1995 (5) + Fall 1995 (58) | 79 |
|  |  |  |  |  |
|  | Lake Ulvenvann | Mature | Fall/Spring 1994/1995 (17) + Spring/Summer 1995 (12) | 29 |
|  |  | Immature | Fall/Spring 1994/1995 (6) | 6 |
|  |  |  |  |  |
| Females | Sandågrotta cave | Mature | Fall/Spring 1994/1995 (55) + Spring/Summer 1995 (44) + Fall 1995 (45) | 144 |
|  |  | Immature | Fall/Spring 1994/1995 (12) + Spring/Summer 1995 (13) + Fall 1995 (28) | 53 |
|  |  |  |  |  |
|  | Lake Lille Lauarvann | Mature | Fall/Spring 1994/1995 (71) + Spring/Summer 1995 (36) + Fall 1995 (91) | 198 |
|  |  | Immature | Fall/Spring 1994/1995 (16) + Spring/Summer 1995 (5) + Fall 1995 (58) | 79 |
|  |  |  |  |  |
|  | Lake Ulvenvann | Mature | Fall/Spring 1994/1995 (14) + Spring/Summer 1995 (13) | 17 |
|  |  | Immature | Fall/Spring 1994/1995 (6) | 6 |

* Explanation for season (with different sampling dates):

- Sandågrotta cave: Fall/Spring 1994/1995 (09.10.94, 11.11.94, 14.12.94, 03.02.95, 03.03.95, 31.03.95), Spring/Summer 1995 (26.06.95, 19.07.95), Fall 1995 (17.08.95).
- Lake Lille Lauarvann: Fall/Spring 1994/1995 (30.09.94, 09.10.94, 11.11.94), Spring/Summer 1995 (26.05.95, 26.06.95, 19.07.95), Fall 1995 (17.08.95, 17.09.95, 05.10.95).
- Lake Ulvenvann: Fall/Spring 1994/1995 (01.09.94), Spring/Summer 1995 (10.05.95, 15.06.95).
