# Supplementary material for: Morphology and life history divergence in cave and surface populations of Gammarus lacustris (L.)
Source: PLoS One. 2018 Oct 25;13(10):e0205556. doi: 10.1371/journal.pone.0205556 (PMC6201897; doi:10.1371/journal.pone.0205556)
Supplement: S1 Fig — (DOCX) [file pone.0205556.s009.docx]

**S1 Fig. Body length quantiles.**

Note that location *grotte* = Sandågrotta cave, *Overfl*. = Lake Lille Lauvarvann, and *Ulven* = Lake Ulvenvann. Further, F= female, M=male and U= immature. Also, Kroppslengde (mm) = body length in mm. Kjønn = sex.

**Grotte, Fall**

**Oneway Analysis of Kroppslengde (mm) By Kjønn**

**Quantiles**

| Level | Minimum | 10% | 25% | Median | 75% | 90% | Maximum |
| --- | --- | --- | --- | --- | --- | --- | --- |
| F | 8,7 | 10,81 | 13,35 | 15,1 | 16,975 | 18,19 | 20,9 |
| M | 7,9 | 10,54 | 14,2 | 17,1 | 18,75 | 20,18 | 22,9 |
| U | 5,2 | 6,6 | 7,3 | 7,9 | 8,6 | 14,51 | 18,9 |

**Grotte, Spring**

**Oneway Analysis of Kroppslengde (mm) By Kjønn**

**Quantiles**

| Level | Minimum | 10% | 25% | Median | 75% | 90% | Maximum |
| --- | --- | --- | --- | --- | --- | --- | --- |
| F | 8,9 | 11,4 | 13,75 | 15,35 | 16,4 | 17,05 | 18,7 |
| M | 7,6 | 11 | 14,575 | 17,05 | 19,375 | 20,34 | 22,2 |
| U | 6,3 | 6,38 | 6,55 | 6,9 | 7,75 | 12,92 | 14 |

**Overflat, Fall**

**Oneway Analysis of Kroppslengde (mm) By Kjønn**

**Quantiles**

| Level | Minimum | 10% | 25% | Median | 75% | 90% | Maximum |
| --- | --- | --- | --- | --- | --- | --- | --- |
| F | 7,2 | 7,93 | 8,5 | 9,85 | 11,6 | 12,4 | 14,6 |
| M | 6,2 | 8 | 9 | 10,2 | 11,3 | 13 | 15,2 |
| U | 4,2 | 5,65 | 6,4 | 7,25 | 8,025 | 9,1 | 11,4 |

**Overflat, Spring**

**Oneway Analysis of Kroppslengde (mm) By Kjønn**

**Quantiles**

| Level | Minimum | 10% | 25% | Median | 75% | 90% | Maximum |
| --- | --- | --- | --- | --- | --- | --- | --- |
| F | 9,3 | 9,77 | 10,725 | 11,4 | 12,125 | 13,57 | 15,9 |
| M | 9,1 | 11,4 | 11,9 | 12,5 | 12,9 | 13,3 | 14,2 |
| U | 3,8 | 3,8 | 3,85 | 4,2 | 4,8 | 4,9 | 4,9 |

**Ulven, Fall**

**Oneway Analysis of Kroppslengde (mm) By Kjønn**

**Quantiles**

| Level | Minimum | 10% | 25% | Median | 75% | 90% | Maximum |
| --- | --- | --- | --- | --- | --- | --- | --- |
| F | 6,9 | 6,95 | 7,4 | 7,75 | 8,4 | 8,85 | 8,9 |
| M | 6 | 6,4 | 7,05 | 8,8 | 9,2 | 9,34 | 9,5 |
| U | 4,9 | 4,9 | 5,125 | 5,95 | 7,8 | 7,8 | 7,8 |

**Ulven, Spring**

**Oneway Analysis of Kroppslengde (mm) By Kjønn**

**Quantiles**

| Level | Minimum | 10% | 25% | Median | 75% | 90% | Maximum |
| --- | --- | --- | --- | --- | --- | --- | --- |
| F | 10,2 | 10,44 | 11,1 | 12 | 12,8 | 13,36 | 13,4 |
| M | 11,5 | 11,77 | 13,2 | 14,05 | 14,5 | 14,97 | 15 |
