# Supplementary material for: Morphology and life history divergence in cave and surface populations of Gammarus lacustris (L.)
Source: PLoS One. 2018 Oct 25;13(10):e0205556. doi: 10.1371/journal.pone.0205556 (PMC6201897; doi:10.1371/journal.pone.0205556)
Supplement: S2 Fig — (DOCX) [file pone.0205556.s010.docx]

**S2 Fig. Egg volume and egg number.**

Note that eggant = number of eggs or fecundity, eggvol = egg volume (mm3), and kropp = body length (mm). Black circles = Sandågrotta cave, red circles = Lake Ulvenvann and blue circles = Lake Lille Lauarvann.


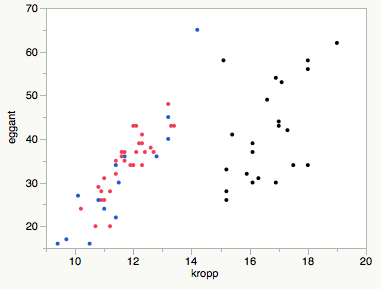

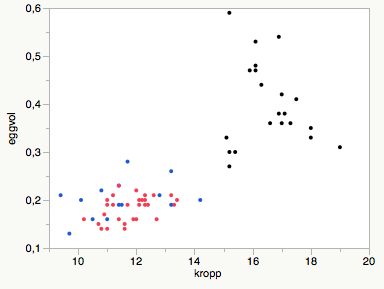


The six individuals that were taken out of the analyses were:

Location Number Date body length egg number stadium eggvolume

Cave 28 26.09.95 14.6 1 4 0.45

Cave 21 19.07.95 15.9 7 6 0.50

Cave 28 19.07.95 14.9 5 6 0.49

Lille Lauar 2 26.05.95 11.2 4 2 0.16

Lille Lauar 8 26.05.95 11.2 6 2 0.15

Lille Lauar 12 26.05.95 11.9 11 3 0.25

Data used for the egg number and egg volume analyses. (locality, egg stage, sampling period, N individuals).

| Locality | Egg stage | Sampling year and month | N individuals for egg number and egg volume |
| --- | --- | --- | --- |
| Sandågrotta cave | 2 | June 1996 (1) | 1 |
|  | 3 | June 1996 (9) | 9 |
|  | 4 | June 1995 (4), June 1996 (8) | 12 |
|  | *Sum* |  | *22* |
|  |  |  |  |
| Lake Lille Lauarvann | 2 | May-July 1995 (4) | 4 |
|  | 3 | May-July 1995 (6), June 1996 (1) | 7 |
|  | 4 | May 1995 (3) | 3 |
|  | *Sum* |  | *14* |
|  |  |  |  |
| Lake Ulvenvann | 2 | May 1995 (13) | 13 |
|  | 3 | May 1995 (17) | 17 |
|  | 4 | May 1995 (1) | 1 |
|  | *Sum* |  | *31* |
