# Supplementary material for: Morphology and life history divergence in cave and surface populations of Gammarus lacustris (L.)
Source: PLoS One. 2018 Oct 25;13(10):e0205556. doi: 10.1371/journal.pone.0205556 (PMC6201897; doi:10.1371/journal.pone.0205556)
Supplement: S3 Fig — (DOCX) [file pone.0205556.s011.docx]

**S3 Fig. Morphological trait quantiles.**

Morphometric analyses boxplot. Note that location *grotte* = Sandågrotta cave, *Overfl*. = Lake Lille Lauvarvann, and *Ulven* = Lake Ulvenvann. Trait codes on y-axes – se main text. Sted = location.

**Raw values:**

**Oneway Analysis of om By Sted**

**Quantiles**

| Level | Minimum | 10% | 25% | Median | 75% | 90% | Maximum |
| --- | --- | --- | --- | --- | --- | --- | --- |
| Grotte | 27,5 | 33,9 | 37,875 | 42,75 | 46,75 | 50,9 | 58 |
| Overfl | 27,5 | 43,5 | 50,125 | 54,5 | 62,25 | 66,05 | 68,5 |
| Ulven | 30 | 34,85 | 37,5 | 44,75 | 51 | 54,2 | 57 |

**Oneway Analysis of oar By Sted**

**Quantiles**

| Level | Minimum | 10% | 25% | Median | 75% | 90% | Maximum |
| --- | --- | --- | --- | --- | --- | --- | --- |
| Grotte | 0,04 | 0,057 | 0,08 | 0,09 | 0,1 | 0,113 | 0,13 |
| Overfl | 0,04 | 0,06 | 0,07 | 0,09 | 0,1075 | 0,13 | 0,14 |
| Ulven | 0,04 | 0,04 | 0,05 | 0,065 | 0,09 | 0,1 | 0,11 |

**Oneway Analysis of ant1 By Sted**

**Quantiles**

| Level | Minimum | 10% | 25% | Median | 75% | 90% | Maximum |
| --- | --- | --- | --- | --- | --- | --- | --- |
| Grotte | 4,7 | 5,77 | 7,225 | 8,7 | 9,825 | 11,29 | 12,3 |
| Overfl | 3,2 | 3,9 | 4,3 | 5,05 | 5,8 | 6,4 | 6,8 |
| Ulven | 2,57 | 3,039 | 3,4325 | 4,28 | 5,1125 | 5,85 | 6,36 |

**Oneway Analysis of ledd1 By Sted**

**Quantiles**

| Level | Minimum | 10% | 25% | Median | 75% | 90% | Maximum |
| --- | --- | --- | --- | --- | --- | --- | --- |
| Grotte | 17 | 19,4 | 23,75 | 27,5 | 31 | 36 | 38 |
| Overfl | 14 | 16,9 | 19 | 21 | 23 | 24,1 | 27 |
| Ulven | 13 | 14,9 | 16,25 | 19,5 | 21,75 | 23 | 24 |

**Oneway Analysis of peds1 By Sted**

**Quantiles**

| Level | Minimum | 10% | 25% | Median | 75% | 90% | Maximum |
| --- | --- | --- | --- | --- | --- | --- | --- |
| Grotte | 1,19 | 1,655 | 1,925 | 2,23 | 2,555 | 2,756 | 3,21 |
| Overfl | 1,1 | 1,285 | 1,375 | 1,545 | 1,73 | 1,953 | 2,19 |
| Ulven | 0,95 | 1,056 | 1,1875 | 1,44 | 1,7075 | 2,031 | 2,31 |

**Oneway Analysis of fl1 By Sted**

**Quantiles**

| Level | Minimum | 10% | 25% | Median | 75% | 90% | Maximum |
| --- | --- | --- | --- | --- | --- | --- | --- |
| Grotte | 3,51 | 3,955 | 5,11 | 6,47 | 7,2825 | 8,578 | 9,29 |
| Overfl | 2,1 | 2,569 | 2,8875 | 3,51 | 4,16 | 4,403 | 4,82 |
| Ulven | 1,62 | 1,999 | 2,23 | 2,85 | 3,3925 | 3,765 | 4,21 |

**Oneway Analysis of ant2 By Sted**

**Quantiles**

| Level | Minimum | 10% | 25% | Median | 75% | 90% | Maximum |
| --- | --- | --- | --- | --- | --- | --- | --- |
| Grotte | 1,7 | 2,87 | 3,65 | 4,5 | 6 | 6,4 | 7,2 |
| Overfl | 1,8 | 2 | 2,3 | 2,7 | 3,575 | 4 | 4,4 |
| Ulven | 1,41 | 1,65 | 1,885 | 2,34 | 3,15 | 3,884 | 4,36 |

**Oneway Analysis of ledd2 By Sted**

**Quantiles**

| Level | Minimum | 10% | 25% | Median | 75% | 90% | Maximum |
| --- | --- | --- | --- | --- | --- | --- | --- |
| Grotte | 8 | 9,7 | 11 | 12 | 16 | 17 | 18 |
| Overfl | 5 | 6 | 8 | 9 | 10 | 11 | 12 |
| Ulven | 6 | 7 | 7 | 8 | 11 | 12 | 13 |

**Oneway Analysis of peds2 By Sted**

**Quantiles**

| Level | Minimum | 10% | 25% | Median | 75% | 90% | Maximum |
| --- | --- | --- | --- | --- | --- | --- | --- |
| Grotte | 1,13 | 1,696 | 2,165 | 2,545 | 3,33 | 3,713 | 4,01 |
| Overfl | 1 | 1,28 | 1,42 | 1,73 | 2,18 | 2,626 | 2,93 |
| Ulven | 0,81 | 0,93 | 1,0275 | 1,31 | 1,7775 | 2,222 | 2,5 |

**Oneway Analysis of fl2 By Sted**

**Quantiles**

| Level | Minimum | 10% | 25% | Median | 75% | 90% | Maximum |
| --- | --- | --- | --- | --- | --- | --- | --- |
| Grotte | 0,57 | 1,174 | 1,495 | 2,02 | 2,71 | 2,883 | 3,27 |
| Overfl | 0,57 | 0,718 | 0,81 | 0,98 | 1,31 | 1,474 | 1,78 |
| Ulven | 0,6 | 0,729 | 0,825 | 1,02 | 1,3725 | 1,692 | 1,93 |

**Log-transformed values:**

**Oneway Analysis of lnom By Sted**

**Quantiles**

| Level | Minimum | 10% | 25% | Median | 75% | 90% | Maximum |
| --- | --- | --- | --- | --- | --- | --- | --- |
| Grotte | 3,31 | 3,522 | 3,635 | 3,755 | 3,845 | 3,928 | 4,06 |
| Overfl | 3,31 | 3,77 | 3,9125 | 4 | 4,135 | 4,191 | 4,23 |
| Ulven | 3,4 | 3,555 | 3,62 | 3,805 | 3,93 | 3,994 | 4,04 |

**Oneway Analysis of lnoar By Sted**

**Quantiles**

| Level | Minimum | 10% | 25% | Median | 75% | 90% | Maximum |
| --- | --- | --- | --- | --- | --- | --- | --- |
| Grotte | -3,2 | -2,919 | -2,4975 | -2,375 | -2,27 | -2,16 | -2,04 |
| Overfl | -3,12 | -2,84 | -2,6 | -2,415 | -2,2525 | -2,058 | -1,99 |
| Ulven | -3,3 | -3,196 | -2,93 | -2,715 | -2,435 | -2,31 | -2,2 |

**Oneway Analysis of lnant1 By Sted**

**Quantiles**

| Level | Minimum | 10% | 25% | Median | 75% | 90% | Maximum |
| --- | --- | --- | --- | --- | --- | --- | --- |
| Grotte | 1,55 | 1,754 | 1,98 | 2,16 | 2,2825 | 2,426 | 2,51 |
| Overfl | 1,16 | 1,36 | 1,46 | 1,62 | 1,76 | 1,86 | 1,92 |
| Ulven | 0,94 | 1,11 | 1,23 | 1,455 | 1,635 | 1,762 | 1,85 |

**Oneway Analysis of lnledd1 By Sted**

**Quantiles**

| Level | Minimum | 10% | 25% | Median | 75% | 90% | Maximum |
| --- | --- | --- | --- | --- | --- | --- | --- |
| Grotte | 2,83 | 2,967 | 3,17 | 3,315 | 3,43 | 3,58 | 3,64 |
| Overfl | 2,64 | 2,824 | 2,94 | 3,04 | 3,14 | 3,184 | 3,3 |
| Ulven | 2,56 | 2,703 | 2,785 | 2,97 | 3,0775 | 3,14 | 3,18 |

**Oneway Analysis of lnpeds1 By Sted**

**Quantiles**

| Level | Minimum | 10% | 25% | Median | 75% | 90% | Maximum |
| --- | --- | --- | --- | --- | --- | --- | --- |
| Grotte | 0,18 | 0,501 | 0,6575 | 0,8 | 0,9375 | 1,013 | 1,17 |
| Overfl | 0,09 | 0,256 | 0,3225 | 0,435 | 0,55 | 0,671 | 0,79 |
| Ulven | -0,05 | 0,056 | 0,175 | 0,365 | 0,5375 | 0,71 | 0,84 |

**Oneway Analysis of lnfl1 By Sted**

**Quantiles**

| Level | Minimum | 10% | 25% | Median | 75% | 90% | Maximum |
| --- | --- | --- | --- | --- | --- | --- | --- |
| Grotte | 1,25 | 1,378 | 1,635 | 1,865 | 1,985 | 2,15 | 2,23 |
| Overfl | 0,74 | 0,94 | 1,0625 | 1,255 | 1,4225 | 1,483 | 1,57 |
| Ulven | 0,48 | 0,69 | 0,805 | 1,05 | 1,225 | 1,324 | 1,44 |

**Oneway Analysis of lnant2 By Sted**

**Quantiles**

| Level | Minimum | 10% | 25% | Median | 75% | 90% | Maximum |
| --- | --- | --- | --- | --- | --- | --- | --- |
| Grotte | 0,53 | 1,051 | 1,295 | 1,5 | 1,79 | 1,86 | 1,97 |
| Overfl | 0,59 | 0,69 | 0,83 | 0,99 | 1,2725 | 1,39 | 1,48 |
| Ulven | 0,34 | 0,5 | 0,6325 | 0,85 | 1,15 | 1,361 | 1,47 |

**Oneway Analysis of lnledd2 By Sted**

**Quantiles**

| Level | Minimum | 10% | 25% | Median | 75% | 90% | Maximum |
| --- | --- | --- | --- | --- | --- | --- | --- |
| Grotte | 2,08 | 2,27 | 2,4 | 2,48 | 2,77 | 2,83 | 2,89 |
| Overfl | 1,61 | 1,79 | 2,08 | 2,2 | 2,3 | 2,4 | 2,48 |
| Ulven | 1,79 | 1,95 | 1,95 | 2,08 | 2,4 | 2,48 | 2,56 |

**Oneway Analysis of lnpeds2 By Sted**

**Quantiles**

| Level | Minimum | 10% | 25% | Median | 75% | 90% | Maximum |
| --- | --- | --- | --- | --- | --- | --- | --- |
| Grotte | 0,12 | 0,525 | 0,7775 | 0,935 | 1,2025 | 1,314 | 1,39 |
| Overfl | 0 | 0,24 | 0,35 | 0,55 | 0,78 | 0,964 | 1,07 |
| Ulven | -0,21 | -0,07 | 0,0275 | 0,27 | 0,5725 | 0,796 | 0,92 |

**Oneway Analysis of lnfl2 By Sted**

**Quantiles**

| Level | Minimum | 10% | 25% | Median | 75% | 90% | Maximum |
| --- | --- | --- | --- | --- | --- | --- | --- |
| Grotte | -0,56 | 0,164 | 0,4025 | 0,705 | 1 | 1,06 | 1,19 |
| Overfl | -0,55 | -0,326 | -0,21 | -0,02 | 0,27 | 0,392 | 0,58 |
| Ulven | -0,51 | -0,312 | -0,1925 | 0,02 | 0,3175 | 0,522 | 0,66 |
